# Engineered cell-degradable poly(2-alkyl-2-oxazoline) hydrogel for epicardial placement of mesenchymal stem cells for myocardial repair

**DOI:** 10.1016/j.biomaterials.2020.120356

**Published:** 2021-02

**Authors:** Yaqi You, Kazuya Kobayashi, Burcu Colak, Piaopiao Luo, Edward Cozens, Laura Fields, Ken Suzuki, Julien Gautrot

**Affiliations:** a Institute of Bioengineering, Queen Mary, University of London, Mile End Road, London, E1 4NS, UK; b School of Engineering and Materials Science, Queen Mary, University of London, Mile End Road, London, E1 4NS, UK; c William Harvey Research Institute, Barts and the London School of Medicine and Dentistry, Queen Mary University of London, EC1M 6BQ, UK

**Keywords:** Poly(2-alkyl-2-oxazoline), Thiol-ene coupling, Engineered hydrogels, Stem cell delivery, Pro-angiogenic, Cardiac repair

## Abstract

Epicardial placement of mesenchymal stromal cells (MSCs) is a promising strategy for cardiac repair post-myocardial infarction, but requires the design of biomaterials to maximise the retention of donor cells on the heart surface and control their phenotype. To this end, we propose the use of a poly(2-alkyl-2-oxazoline) (POx) derivative, based on 2-ethyl-2-oxazoline and 2-butenyl-2-oxazoline. This POx polymer can be cured rapidly (less than 2 min) via photo-irradiation due to the use of di-cysteine cell degradable peptides. We report that the cell-degradable properties of the resulting POx hydrogels enables the regulation of cell protrusion in corresponding 3D matrices and that this, in turn, regulates the secretory phenotype of MSCs. In particular, the expression of pro-angiogenic genes was upregulated in partially cell-degradable POx hydrogels. Improved angiogenesis was confirmed in an *in vitro* microfluidic assay. Finally, we confirmed that, owing to the excellent tissue adhesive properties of thiol-ene crosslinked hydrogels, the epicardial placement of MSC-loaded POx hydrogels promoted the recovery of cardiac function and structure with reduced interstitial fibrosis and improved neovascular formation in a rat myocardial infarction model. This report demonstrates that engineered synthetic hydrogels displaying controlled mechanical, cell degradable and bioactive properties are particularly attractive candidates for the epicardial placement of stem cells to promote cardiac repair post myocardial infarction.

## 1 Introduction

Myocardial infarction (MI) remains a major cause of death and disability worldwide. Mesenchymal stromal cell (MSC)-based therapy is an emerging treatment for MI. The accepted therapeutic mechanism associated with MSC-promoted cardiac repair is the paracrine signalling: beneficial secretions from MSCs are able to induce tissue repair of the damaged myocardium. Particularly, epicardial placement of MSCs has recently been successful in pre-clinical studies. This approach achieves higher retention rates of donor cells, compared to other methods, which leads to enhanced myocardial repair and cardiac function post-MI [1]. In order to translate such pre-clinical success into clinically effective strategies, several hurdles remain to be tackled. These include the type and quality of donor MSCs and the design of biomaterials enabling efficient epicardial placement whilst regulating stem cell phenotype [[2], [3], [4], [5]].

Recently, amnion-derived MSCs have been proposed as an effective and convenient source for cell therapy. Ishikane et al. placed rat foetal membrane-derived MSC sheets on the epicardium in a chronic MI rat model [6,7]. They observed a clear improvement in cardiac function and a particularly strong induction of angiogenesis in the ischemic area, an important factor contributing to tissue regeneration. Suzuki et al. investigated the epicardial placement of amnion-derived MSCs in an MI rat model (ischemic cardiomyopathy) via the self-assembling peptide hydrogel PuraMatrix [8]. They reported excellent retention of the donor cells and a clear improvement of cardiac function, compared to intramyocardial injection (IM) methods. However, the low pH associated with PuraMatrix hydrogels is a disadvantage of this approach. This highlighted the importance of hydrogel design for epicardial placement. More recently, they have developed another approach for the epicardial placement of amnion-derived MSCs using a fibrin sealant film [9]. This strategy also resulted in improved therapeutic effects compared to IM injection, including the enhancement of neovascularization and decreased pathological fibrosis in association with myocardial upregulation of genes implicated in cardiac repair. Overall, strategies for epicardial placement that do not result in changes in local pH and that are based on mechanically stable biomaterials, whilst promoting the pro-angiogenic secretory phenotype of MSCs are still required.

The engineering of biomaterials enabling the simple delivery and efficient retention of MSCs, and allowing to enhance their therapeutic function and survival rate after transplantation is essential to the success of stem cell-based cardiac repair procedures. Several strategies, including prefabricated patches, have been proposed for MSC delivery to the epicardium [[10], [11], [12], [13]]. However, production of these patches required additional cell culture (isolated from patients) and/or patch production procedure prior to transplantation, therefore requiring suture or glue for stabilisation at the heart surface. Compared to prefabricated patches, *in situ* formation of MSC-loaded hydrogels (potentially from allogenic sources) that spontaneously adhere and set on the heart surface following epicardial placement offers important advantages in terms of simplification of procedures and reduction of costs (by avoiding expensive *in vitro* cell culture and making use of inexpensive biomaterials). In addition, *in situ* formed MSC-loaded hydrogels will enable enhanced integration to the cardiac surface and myocardium [8]. This should result in encapsulation, preventing the shedding of donor MSCs and migration to extra-cardiac tissues [9]. Finally, cardiac repair will be enhanced by the design of hydrogels able to regulate cell phenotype (in particular the secretion of reparative cytokines and growth factors).

A wide variety of hydrogels have been proposed for cell encapsulation and delivery. Poly (ethylene glycol) (PEG) based systems have attracted significant attention due to the inherent hydrophilicity of this scaffold and its protein-resistance [[14], [15], [16]]. Peptide-crosslinked PEG hydrogels enable the regulation of cell-based degradability and can be functionalised with additional bioactive peptides promoting cell adhesion [17,18]. Although PEG is generally considered as a bioinert polymer, a number of recent reports indicated that it could elicit immune responses, perhaps owing to repeated sensitisation [19,20]. PEG also offers limited degrees of functionalisation and typically requires star-shape polymer structure design, synthetically more challenging, or the introduction of functional moieties directly in the polymer backbone, often difficult to control in copolymerisation with ethylene oxide. This limited structural design flexibility can prevent the control of mechanics over a wide range of polymer content, compared to polymer structures bearing side chain functionalities. Other approaches have been proposed for the design of synthetic hydrogels, for example based on modified hyaluronic acid polymers [[21], [22], [23]] or gelatin [[24], [25], [26]]. Overall, it is now clearer that suitable hydrogel design (e.g. with controlled mechanics, degradability and promoting cell adhesion) can enable the regulation of stem cell phenotype [27].

Fibrin glues have been widely used for epicardial placement of stem cells. It was found to result in increased donor cell engraftment, compared to simple IM injection [28]. This led to an enhancement in donor cell retention and improved recovery of cardiac function. Similarly, bi-layers of collagen and fibrin gels were applied to deliver rat amnion-derived MSCs to the epicardium in a rat ischemic cardiomyopathy model [9]. This approach enhanced donor cell engraftment compared to intramyocardial injection and enhanced cardiac function recovery. This was found to be associated with enhanced neovascularization and decreased fibrosis of the damaged myocardium. However, the engineering of the adhesive, mechanical and degradative properties of fibrin and collagen gels remains relatively limited. Other gels that have been used for delivery of amnion-derived MSCs include self-assembling peptide hydrogels such as PuraMatrix [8]. Epicardial placement of foetal membrane-derived MSCs in an ischemic cardiomyopathy rat model, using such self-assembled peptides, led to the upregulation of several genes associated with recovery of cardiac functions, including HIF1-α, IL-10, TIMP-1, MMP-2, IGF-1, CXC12 and FGF-2. However, the requirement for careful optimisation of the hydrogel pH prior to injection was found to limit the use of such approach in the clinic, although the adhesion to the epicardium was found to be satisfactory.

Recently, poly(2-alkyl-2-oxazolines) (POx) have emerged as attractive alternatives to PEG for the design of hydrogels and “smart” biomaterials, for applications such as drug delivery, membrane technologies, surface coatings, vectors for gene delivery, stimuli responsive systems and biosensors [[29], [30], [31]]. Indeed, poly(2-methyl-2-oxazoline) (PMeOx) and poly(2-ethyl-2-oxazoline) (PEtOx) display similar cytocompatibility, hydrophilicity, solution conformation and viscosity to PEG, but can be conveniently functionalised with pendent moieties enabling their biofunctionalisation and crosslinking [32]. This suggested that POx could be an ideal backbone for the formulation of hydrogels and other crosslinked networks for application in the biomedical field [33,34].

POx hydrogels, based on the crosslinking of POx backbones presenting alkene side chains, have been developed for the 3D encapsulation of fibroblasts [35,36]. For example, a poly(2-methyl-2-oxazoline)-co-(dec-9-enyl-2-oxazoline) polymer backbone was crosslinked with dithiothreitiol (DTT) to form hydrogels under UV irradiation (365 nm), via thiol-ene coupling. Fibroblasts were photo-encapsulated in such 3D POx gels in the presence of different concentrations of the cell adhesive CRDGSG peptide, which was found to influence cell viability. However, cells remained rounded within 1–8 days of culture presumably due to the high stiffness of the POx gels used (3.4–4.5 kPa) and the lack of degradation sites. It was therefore concluded that thiol-ene crosslinked POx hydrogels is attractive for cell encapsulation, providing the engineering of improved controlled degradation, cell spreading and viability. In particular, the crosslinking of POx backbones with relatively high functionalities (>10 alkene moieties per chain) with accessible di-cysteine peptides can be relatively efficient in mild conditions and induces little toxicity to cells [[37], [38], [39]].

In this work, we developed peptide-crosslinked degradable hydrogels based on poly(2-ethyl-2-oxazoline-co-2-butenyl-2-oxazoline). We studied the impact of formulation on the mechanical properties of the resulting hydrogels and, in turn, the encapsulation and morphology of MSCs. We investigated how such microenvironment regulates the secretory phenotype of MSCs, in particular with respect to proangiogenic factors. We then made use of the excellent tissue adhesive properties of thiol-ene crosslinked POx hydrogels to deliver MSCs to the epicardium of hearts in a rat MI model. We studied the impact of such epicardial placement on neovascularization, tissue fibrosis and the recovery of cardiac function. Our results indicated that the ease with which POx hydrogel formulations can be designed is particularly well-suited to the development of soft scaffolds for tissue engineering.

## 2 Materials and methods

### 2.1 2D cell culture of MSCs

Human bone marrow derived mesenchymal stem cells (bm-MSCs) were purchased from Promocell at Passage 2 and were cultured with MSC Growth Medium 2 (Promocell). Cells were plated in T75 flasks at a density of 4000 cells/cm^2^. Cells were cultured in incubators at 37 °C and 5% CO_2_. Cell culture medium was changed every other day. Cells were passaged when 80–90% confluency was reached, using Accutase solution (Promocell) for detachment. Cells of passage 3 to 6 were used for experiments.

Human amnion-derived mesenchymal stem cells (ha-MSC) were provided by Dr. Kenichi Yamahara [40]. Their marker expression (positive for CD73 and CD29, negative for CD45 and CD34), as well as their ability to differentiate into adipocytes and osteocytes, at least up to passage 20, was previously established in our lab [9]. Cells were defrosted from cryopreservation vials at Passage 10 and seeded in T75 flasks at a density of 10,000 cells/cm^2^, and cultured in aMEM (Gibco) with 10% fetal bovine serum (FBS), supplemented with 1% penicillin and streptomycin and 10% of human basic fibroblast growth factor (bFGF, 10 ng/mL). Cells were cultured in incubator at 37 °C and 5% CO_2_. Cell culture medium was changed every other day. Cells were passaged when at 80%–90% confluency by detachment using 0.25% Trypsin with 0.2% EDTA (Sigma). Passage 11 to 14 cells were used for experiments.

### 2.2 General methods for cell-based assays

#### 2.2.1 Live dead staining and cell viability assays

Cell viability was assessed via a live/dead assay (ThermoFisher). The staining solution was prepared in PBS containing 2 μM calcein AM and 4 μM Ethidium Homodimer-1. The medium was aspirated from the well, replaced with the staining solution and left in the incubator for 20 min before imaging. Images were taken using a Leica DMI 4000B Epifluorescence Microscope (CTR 4000 lamp, 10 X 0.3 NA lens). Cell viability was calculated by counting the number of live cells divided by the total cell number. Images were analyzed using Image J.

#### 2.2.2 Nuclear and actin staining

F-actin was stained by phalloidin (Sigma, P2141) and nuclei were stained by (4′-6-Diamidino-2-phenylindole (DAPI, Sigma) after standard fixation and permeabilization procedures. Samples were washed and incubated with the staining solution (0.25% Gelatin, 10% FBS, 1/500 phalloidin (stock concentration 33.3 μg/mL), and 1/1000 DAPI (stock concentration 5 nM) in PBS) for 1 h at room temperature before imaging. Images were taken by a Zeiss LSM 710 Confocal Microscope (EC Plan-Neofluar20 X/0.5 M27 lens) and analyzed using the ZEN imaging software.

#### 2.2.3 CyQUANT™ DNA quantification for cell proliferation

The CyQUANT™ cell proliferation assay kit (Thermo fisher scientific) was used to quantify DNA content of 3D cell encapsulated hydrogels. Frozen hydrogel samples (after aspiration of medium and washing with PBS), harvested at different time points, were stored in a −80 °C freezer. The samples were thawed and lysed by addition of a buffer containing the CyQUANT™ GR dye, and incubated at room temperature for 1 h before measuring the fluorescence intensity of the supernatant (1 mL CyQUANT™ GR/cell lysis working solution contains 2.5 μL of the CyQUANT™ GR dye stock solution (400 X in DMSO) in nuclease-free distilled water). 200 μL of gel lysis solution was transferred into a black 96-well plate (Corning). The samples were measured using a fluorescence reader at an excitation of 480 nm and emission of 520 nm.

#### 2.2.4 Immunostaining for CD31

After washing with PBS, samples were fixed with 4% PFA for 30 min at room temperature, washed again with PBS and permeabilized with 0.2% Triton X-100 (Sigma- Aldrich) for 5 min, also at room temperature. After washing of these solutions, the samples were incubated in blocking buffer (5% BSA in PBS) for 1 h. Afterwards, samples were incubated in the primary antibody solution (overnight for anti-CD31 Monoclonal Antibody, Thermofisher Scientific, 1: 100, diluted in blocking buffer). After washing, samples were incubated in a solution of Alexa Fluor 488-conjugated secondary antibody for 1 h at room temperature, together with DAPI and phalloidin, diluted in blocking buffer (AF488 antibody, 1 μg/mL, 1:1000 from stock; DAPI, 1:1000 from 5 nM stock; phalloidin, 1:500 from 33.3 μg/mL stock). Samples were washed and images were taken directly without mounting, with a Zeiss LSM710 Confocal Microscope (EC Plan-Neofluar20X/0.5 M27 lens) and analyzed by ZEN imaging system.

### 2.3 Fibrin gel formation for 3D cell culture

Fibrin gels were crosslinked *in situ* by mixing fibrinogen and thrombin solutions. 1 mL of solution A was prepared with fibrinogen (10 or 5 mg/mL), 44 μL collagen solution (stock concentration of 9 mg/mL, resulting in 400 μg/mL solution) and 7.5 μL aprotinin (stock concentration of 40 U/mL), in sterile PBS. Aprotinin was added to reduce hydrogel degradation. 1 mL of solution B was prepared from 40 μL thrombin (stock concentration of 2.5 U/mL, containing 0.1% BSA) in PBS. Solutions A and B were warmed up to 37 °C in a water bath, during which times cells were passaged as described in section 2.1. Cells were counted and pellets of known cell numbers was obtained, after centrifugation. Solution B was mixed with the cell pellet to make up solution C. 50 μL of solution A and 50 μL solution C were mixed in a new Eppendorf tube and immediately transferred into each well of a μ-Slide (8 well plates on microscope glass coverslips, Ibidi). Overall, the composition of a 1 mL fibrin gel is: 2.5 or 5 mg/mL fibrinogen, 0.2 mg collagen and 0.15 U aprotinin and 2 U thrombin. Gels were allowed to form at 37 °C for 5 min before adding growth medium. Gel samples were incubated at 37 °C and 5% CO_2_. Cell culture medium was changed every day.

### 2.4 Synthesis of POX hydrogels

The synthesis of the copolymer (poly (2-ethyl-2-oxazoline-co-2-butenyl-2-oxazoline) (POx) was based on the cationic ring opening polymerization reaction of 20% 2-butenyl-2-oxazoline and 80% 2-ethyl-2-oxazoline monomers [37,41,42]. The resulting poly-2-alkyl-2-oxazoline copolymer contained alkene functional groups and had a M_n_ of 6300 g/mol (corresponding to approximately 7 alkene moieties per chains).

By varying the composition of crosslinkers (degradable vs. non-degradable), hydrogels degrading at different rates were targeted. Details of the composition of the gels studied can be found in Table 1. Stock solutions of the non-degradable poly(ethylene glycol) dithiol (PEGDT, Sigma) and the degradable peptide GCGPQGIAGQGCG (GIA, Proteogenix) were prepared with concentrations of 225 mg/mL and 248 mg/mL, respectively. Two different final concentrations of POx were studied: 20 and 30 wt%, corresponding to 90 and 135 mM of alkene residues. To promote cell adhesion, an RGD (Arg-Gly-Asp) bioactive integrin ligand peptide (GCGYGRGDSPG, Proteogenix, stock solution of 456 mg/mL) was incorporated, also via thiol-ene coupling, during hydrogel formation. To promote photocuring, samples were irradiated with UV light (Omnicure S1500, 365–420 nm, with an intensity of 20 mW/cm^2^) for 120 s for gelation in, the presence of photoinitiator Irgacure 2959 (I2959) (3 mol% with respect to thiols, 250 mg/mL, Sigma).

**Table 1:** Composition of the POx hydrogels studied.

POX Gel	[Alkene] (mM)	[Thiol] (mM)	Thiol/ene ratio	POX (mg/mL)	PEGDT (mg/mL)	GIA (mg/mL)	RGD (mg/mL)	I2959 (mg/mL)
POx1-50	135	67.5	0.5/1	91.1	33.8	37.3	12	0.46
POx2-90	90	81	0.9/1	60.7	40.5	4.5	14.4	0.54
POx2-75	90	81	0.9/1	60.7	30.4	11.2	14.4	0.54
POx2-50	90	81	0.9/1	60.7	20.3	22.4	14.4	0.54
POx2-25	90	81	0.9/1	60.7	10.1	33.5	14.4	0.54
POx2-0	90	81	0.9/1	60.7	0	44.7	14.4	0.54

### 2.5 MSC encapsulation in POx hydrogels

bm-MSCs (P3 to P6) and ha-MSCs (P11 to P14) were suspended in sterilized POx hydrogel precursor solutions at a final cell density of 500,000 cells/mL. This suspension was transferred into μ-slide wells, with a glass bottom (100 μL/well). Cell encapsulation was performed by UV curing using an Omnincure system at 20 mW/cm^2^ for 2 min at room temperature. After gelation, the corresponding MSC growth medium was added covering the top of each well, and incubated for different times prior to further analysis. Cell culture medium was changed every day.

### 2.6 Rheological characterization of hydrogels

A DHR-3 rheometer (TA Instruments) was used for rheological characterization of POx hydrogels. To follow photocuring, i*n situ* photo-rheology was performed using a 250 μm geometry gap (20 mm plate with methacrylate functionalised glass at the top), with 1% oscillatory strain and an oscillation frequency of 1 Hz, at room temperature. 100 μL of gel solution was placed on the quartz plate, the top geometry was lowered to the desired gap and the oscillatory program was started, followed by UV irradiation (Omnicure S1500, 365–420 nm, 20 mW/cm^2^). UV light was turned on between 30 and 150 s following the start of data acquisition in each test to capture the gelation progression.

### 2.7 RNA extraction from 3D gels and quantitative RT–PCR

Cells were encapsulated in 3D hydrogels and cultured for three days. The medium was aspirated, prior to freezing on dry ice. Frozen samples were stored in −80 °C freezer until RNA extraction was started. The trizol method, coupled to the RNeasy Mini Kit (Qiagen) plus DNAse digestion method was used for RNA extraction. Gel samples were transferred to Precellys lysing kit tubes (The science of Lysing, for tissue homogenizing) and 1 mL of trizol reagent (Life technologies) was added to each sample prior to homogenization using a power tissue homogenizer (Precellys). The supernatant was transferred to a new 1.5 mL Eppendorf tube. Trizol manual protocols (Invitrogen) were performed prior to RNA clean up with RNeasy Mini Kit (on column DNAse digestion). RNA was reverse transcribed to cDNA using a high capacity cDNA reverse transcription kit (Applied Biosystems). Quantitative RT-PCR reactions were carried out with PowerUp SYBR Green Master Mix (Applied Biosystems™ A25743). Gene expression assays for human primers of β-2M, MMP-2, SDF-1, VCAM-1, IGF, HGF, VEGF, IL-10, TGF-β, TIMP-1 were tested (Primers were shown in Supplementary Table S1). All data were normalized to the β-2m housekeeping gene expression. Relative quantification of each gene was determined using the 2-ΔΔCT method. Data was analyzed by GraphPad Prism 7.1.

### 2.8 Microfluidic chips for angiogenesis assays

Multichannel microfluidic chips were used for the characterization of angiogenesis in co-cultures of HUVECs (Human Umbilical Vein Endothelial Cells, Promocell; cultured in EBM2 medium) and encapsulated bmMSCs. The geometry of the multi-channel microfluidic chips is shown in Supplementary Fig. S1. This system displays four channels. The dimension for each channel is 700 μm in width. In the central left channel, 10 μL of fibrin pre-gel solution (prepared from 1 mL fibrin gel which contains 5 mg fibrinogen, 0.2 mg collagen, 0.15 U aprotinin and 2 U thrombin) was injected. In the right channel, either 10 μL of fibrin gel or POx gel, or 100,000 bmMSCs cultured in fibrin or POx gels, or conditioned medium collected from bmMSCs cultured in fibrin or POx gels were injected. HUVECs were seeded at the surface of the fibrin gel (central gel channel), from the left medium channel (2000 cells in 10 μL–2.0 M cells/mL). EBM2 (Endothelial cell Basal Medium 2, Promocell) was used in this system as basic growth medium for HUVECs and MSCs, without supplementing with VEGF. Medium was changed twice a day.

### 2.9 Characterization of hydrogel adhesion and mechanical stability upon tissue extension

#### 2.9.1 Shear lap test for the characterization of gel adhesion to the epicardium

Porcine heart tissues were dissected from the left ventricle of pig hearts into 1.5 × 1.5 cm samples. Heart tissues were mounted on a lap shear rig (Poly (methyl methacrylate, PMMA slides). 100 μL gel solution was pipetted directly on the heart surface, covered by a microscopy glass slide and cured under UV light. The coverslips were plasma treated to promote strong bonding with the gel, therefore enabling probing of the tissue-gel interface. Shear lap tests were performed using an Instron mechanical tester (Instron 5967) fitted with a 100 N load cell. Initially the samples were loaded into the lower clamp. The upper clamp was then carefully lowered and tightened on the upper PMMA slide. The contact area between the porcine cardiac sample and plasma treated glass slide was measured with a caliper prior to clamping. The samples were then pulled apart at a constant rate of 3 mm/min. The maximum load was then extracted from the data and this was used to calculate the adhesive shear strength using the following formula. Shear strength (Pa) = (maximum load (N))/(contact area (m^2^)).

#### 2.9.2 Characterisation of gel extension and fracture upon stretching of the epicardium

Heart tissues were dissected from the left ventricle of pig heart into 3 × 1.5 cm samples. 100 μL gel solutions (containing 5 mol % of an FITC-labelled peptide; GCGGRGESP-(Lys-FITC)-G) were pipetted directly onto the heart surface and cured under UV light for 2 min (Omnicure S1500, 365–420 nm light source, 20 mW/cm^2^). Uniaxial extension was applied to the resulting samples using an Instron mechanical tester (Instron 5967) fitted with a 100 N load cell. Tissue samples coated with cured gels were placed in the clamps of the Instron stretched at a constant rate of 3 mm/min. The initial dimension of epicardium tissues was measured with a caliper and the strain induced upon stretching was recorded. The experiment was stopped when the tissue had been stretched to 50% strain and the morphology of the FITC labelled gels was inspected by epifluorescence microscopy, using a Leica DMI 4000B Epifluorescence Microscope (CTR 4000 lamp, 10 × 0.3 NA lens).

### 2.10 Epicardial placement of MSC-loaded POx hydrogels in a rat MI model

All studies were performed with the approval of the institutional ethics committee at Queen Mary University of London and the Home Office, UK. The animal investigations conform to the Principles of Laboratory Animal Care formulated by the National Society for Medical Research and the Guide for the Care and Use of Laboratory Animals (United States National Institutes of Health Publication, 1996).

#### 2.10.1 Induction of MI in rats

Acute myocardial infarction (AMI) rats model were induced in male Lewis rats (body weight near 300 g, Charles River, UK). Following left thoracotomy under isoflurane anesthesia and mechanical ventilation (Havard Apparatus, UK), the left anterior descending artery (LAD) was ligated 1–1.5 mm from its origin with 6-0 silk suture. After MI induction, POx gels or POx gels encapsulating human amnion derived MSCs were applied on the epicardium and cured under UV light for 2 min (Omnicure S1500, 365–420 nm, 20 mW/cm^2^). Finally, the chest and skin were sutured.

#### 2.10.2 POx gel curing process optimisation in an MI rat model

To test the gel curing process and gel retention in realistic conditions prior to surgery, FITC labelled POx gels (Pox2-75 gels containing 5 mol% FITC-labelled peptide GCGGRGESP-(Lys-FITC)-G) were cured on the infarct area of epicardium of rat hearts (Omnicure S1500, 365–420 nm light source, 20 mW/cm^2^). To improve the retention of the uncrosslinked polymer mixture, we used a cylinder to constrain the injected fluid and curing was carried out in two stages: 15 s in the injection pipette tip and 120 s after injection on the epicardium.

Human amnion-derived mesenchymal stem cells (haMSCs) were used for *in vivo* studies. Cells were cultured in T75 flasks and then passaged at 85–90% confluence. CM-Dil (Thermo Fisher Scientific) was used as red tracker for cells at a 1:200 dilution (5 μM), following a published protocol [43]. After induction of MI in the rat model, the gel-cell precursor mixtures (100 μL containing 2 million cells) were directly pipetted onto the infarcted area of the epicardium (15 s pre-curing in the pipette tip, followed by 2 min after pipetting on the epicardium). After the gel was formed, the heart, including the gel layer, was recovered and stored in OCT (VWR International, cooled with liquid nitrogen) and transferred to a −80 °C freezer for storage and further analysis.

#### 2.10.3 *In vivo* epicardial placement in MI rat models

30 male Lewis rats (body weight 383.9 ± 22 g, Charles River, UK) were applied in *in vivo* experiments. Three groups were selected: 1. myocardial infarction induction only (MI, N = 10); 2. MI induction followed by POx gels placement (MI + Gel, N = 10); 3. MI induction followed by POx gel encapsulating ha-MSCs (MI + Gel + MSC, N = 10). The acute MI model was created as described in section 2.10.1. For the MI group, the chest and skin were sutured immediately after MI induction. For the MI + Gel group, after MI induction, 200 μL of POx2-75 gel was applied on the epicardium followed by UV curing. 15 s of UV curing was performed in the pipette tip, before the gel precursor was applied on the epicardium surface to increase the viscosity of the gel precursor. The UV curing time post-application to the epicardium was 105 s. The chest and skin were then closed and sutured. For MI + Gel + MSC group, after MI induction, 200 μL of POx2-75 loaded with 2 million ha-MSCs (N = 5), or 2 million CM-Dil labelled ha-MSCs (N = 5) were cured on the epicardium and cured following the two step protocol used for the MI + Gel group. The chest (6-0 braided silk suture) and skin (4-0 Vicryl (polyglactin 910) for suturing the skin) were then carefully closed without disturbing the gel-cell layer and sutured. All rats were kept and observed for 4 weeks post-surgery. The schematic process of the *in vivo* study is shown in Supplementary Fig. S2.

#### 2.10.4 Echocardiography for quantification of cardiac function measurement

Transthoracic echocardiography was performed on rat models at day 28 post-surgery using the Vevo-770 high-resolution echocardiography imaging system (Visual Sonics), with rats under isoflurane anesthesia. Several parameters including heart rate, left ventricle end-diastolic dimension (LVDd) and left ventricle end-systolic dimension (LVDs) were measured under M-Mode and B-mode. Left ventricular ejection fraction (LVEF) and left ventricular fraction shortening (LVFS) were calculated from the data obtained with 2-dimensional tracing. All data were collected blind from three measurements.

#### 2.10.5 Cryo-section, immunostaining and histological analysis

Cryo-sections of cardiac samples were cut from the apex and base separately from left ventricles, with a thickness of 6 μm. Sections were fixed with 4% PFA for 30 min at room temperature and then incubated in blocking buffer (5% BSA in PBS) for 30 min while shaking. For α-sarcomeric actin staining, sections were incubated overnight with the primary antibody against α-sarcomeric actin (α-SA; Thermo Fisher EA-53; 1:100). After washing, the secondary antiboday goat anti-mouse IgG (H + L; Alexa Fluor® 488, ThermoFisher, 1:300), as well as DAPI (1:500 dilution from 5 nM stock) were applied and incubated for 30 min at room temperature before washing and mounting on glass slides. For Isolectin B4 staining, sections were incubated overnight with Isolectin B4 (Vector Laboratories, 1:100). After washing, streptavidin (Alexa Fluor® 488 conjugate, ThermoFisher, 1:300), as well as DAPI (1:500 dilution from 5 nM stock) were applied and incubated for 30 min at room temperature before washing and mounting on glass slides. Images were acquired by fluorescence microscopy (BZ8000; Keyence) at different magnifications to evaluate gel-cell layer retention and specific markers for donor cells and capillary density.

To quantify capillary densities, images stained with Isolectin B4 were taken from 5 fields of each heart section from border areas (peri-infarct areas directly surrounding the infarct without obvious cardiomyocytes loss) at 20× magnification. The number of capillary was calculated using Image J (Threshold followed by particle analysis). Capillary densities were defined as capillary number per mm^2^.

#### 2.10.6 Picrosirius red staining

Frozen heart cryo-section slides were fixed with 4% PFA for 30 min at room temperature and then washed with distilled water 3 times. Slides were then dried at room temperature and immersed in 1.5% phosphomolybdic acid for 30 min. After washing with distilled water 3 times, slides were immersed in 0.1% Sirius Red for 45 min. The slides were then washed and immersed in 0.5% acetic acid for 3 min. The samples were dehydrated by 70% ethanol for 20 s once and 100% ethanol for 20 s twice. Then samples were dehydrated by Xylene for 20 s twice. After the samples dried, DPX mounting medium was applied on the surface of slides and coverslips were mounted on top and subsequently imaged. Images were taken by fluorescence microscopy (BZ8000; Keyence) at 20× magnification. To quantify the collagen fraction, 5 independent fields from infarct areas, border areas and remote areas were analyzed for each heart section. Quantification was carried out using Image J. Collagen fraction (%) was defined as staining area/total area × 100%. Wall thickness and infarct size were quantified using Picrosirius staining images by Image J. Wall thickness was calculated from 3 sections from infarct areas. Infarct size was defined as size of infarct area/size of left ventricle area × 100%. Infarct size was measured as the ratio of both epicardial and endocardial scar lengths relative to total epicardial and endocardial circumference as previously described [44].

#### 2.10.7 Quantitative assessment of donor cell retention

To analyze the retention of donor ha-MSCs 28 days post-implantation, samples of whole left ventricle were collected and genomic DNA was extracted using AllPrep DNA/RNA Mini Kit (Qiagen) according to the steps of DNA isolation. Total DNA yield including rat DNA and human DNA were measured by Nanodrop. Human specific gene of ALU were detected by PCR. The expression of ALU was normalized to the housekeeping gene GAPDH. To generate a standard curve, the left ventricle samples from MI only samples were mixed with known HAMSC numbers (1 × 10^6^, 1 × 10^5^, 1 × 10^4^, 1 × 10^3^, 1 × 10^2^). Donor cell survival number and survival rate were calculated according to the standard curve (Supplementary Fig. S8).

#### 2.10.8 Gene expression within rat cardiac tissues

Total RNA was extracted from heart samples of whole left ventricle after 28 days implantation using AllPrep DNA/RNA Mini Kit (Qiagen) following the RNA isolation protocol proposed in the Mini Kit. The RNA was reverse transcribed to cDNA using the high capacity cDNA reverse transcription kit (Applied Biosystems). Sybergreen primers for rat genes, including MMP-2, SDF-1, VCAM-1, IGF, HGF, VEGF, IL-10, TGF-β, TIMP were detected by quantitative RT-PCR, carried out with PowerUp SYBR Green Master Mix (Applied Biosystems™). All data was normalized to β-2M housekeeping gene expression. Relative quantification of each gene was determined using the 2-ΔΔCT method. Data was analyzed by GraphPad Prism 7.1.

### 2.11 Data analysis and statistics

Data analysis and Tukey's *t*-test (one way ANOVA test) were performed in Origin 8.5 (*p < 0.05, ** 0.001 < P < 0.05, ***p < 0.001, ****p < 0.0001). The gene expression data was analyzed for statistical significance using GraphPad Prism 7.1. Unless otherwise noted, all experiments were carried out in three independent replicates. All data presented are reported as mean ± SEM.

## 3 Results

### 3.1 Synthesis and mechanical properties of cell-degradable POx hydrogels

**Fig. 1:**
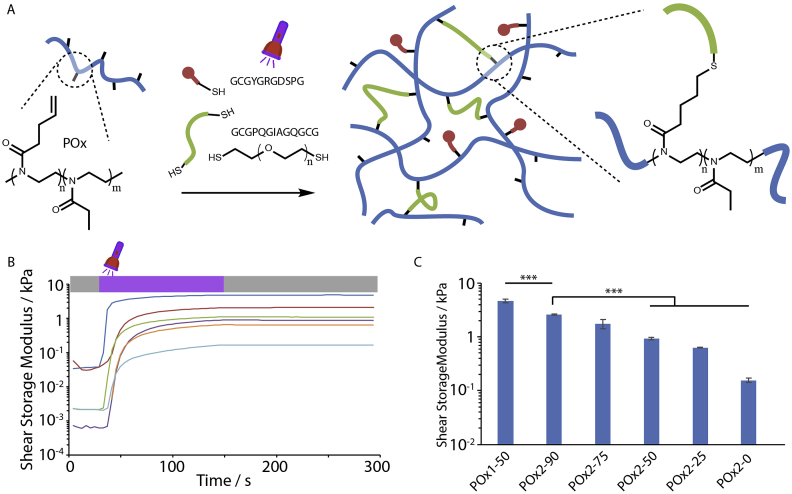
Poly(2-ethyl-2-oxazoline) hydrogel assembly and mechanical properties. A. Schematic representation of POx hydrogel assembly and chemical structure. Polymer backbone (blue) combined with crosslinking GIA di-cysteine peptides/PEG dithiol (green) and cell-adhesive RGD peptides (red). B. Impact of the crosslinker composition (PEG/GIA) on gelation of POx hydrogels. Photo-rheology experiments indicating that shear storage moduli reach a plateau after 120 s of photoirradiation. Experimental conditions: POx concentration: 91.1 or 60.7 mg/mL; thiol/alkene ratio of 0.5/1 or 0.9/1, 10 mol% RGD, 3 mol% I2959 photoinitiator and 120 s UV exposure (see Table 1 for details of compositions; mol% correspond to the total moles of alkene, apart from the initiator, which corresponds to the total moles of thiols). Blue, POx1-50; red, POx2-90; green, POx2-75; purple, POx2-50; orange, POx2-25; turquoise, POx2-0. C. Corresponding shear storage moduli extracted from frequency sweeps at a frequency of 1 Hz and a strain of 0.4% (errors shown are standard errors from three independent measurements). ***p < 0.001. (For interpretation of the references to colour in this figure legend, the reader is referred to the Web version of this article.)

The copolymer (poly(2-ethyl-2-oxazoline-co-2-butenyl-2-oxazoline) (POx) was synthesised via the cationic ring opening polymerization of the corresponding 2-alkyl-2-oxazolines [37]. This polymer was then used as backbone for crosslinking with dithiols such as poly(ethylene glycol) dithiol (PEGDT) and the cell-degradable GIA di-cysteine peptide (GCGPQGIAGQGCG, Fig. 1A). To promote cell adhesion, a monocystein RGD peptide (GCGYGRGDSPG) was introduced into this system. In order to control the degradation rate of POx hydrogels, we introduced different ratios of PEGDT vs. GIA peptide crosslinker (Table 1; for the nomenclature used, the index next to POxA reflects the concentration of the polymer backbone, whereas the number appearing after the dash, as in Pox2-X, indicates the composition in mol% of non-degradable PEGDT). In order to investigate how such changes impacted the curing and mechanical properties of the resulting hydrogels, we carried photo-rheology experiments (Fig. 1B and C). After a short period of equilibration of the system, the evolution of the storage (G′) and loss shear moduli (G″) were monitored during and post UV exposure. Upon UV exposure, for a duration of 120 s, the storage modulus increased rapidly in the first 20 s and reached a plateau by 120 s of exposure (Fig. 1B). The concentration of the POx backbone had a significant impact on the storage modulus, increasing at higher polymer contents, in agreement with other synthetic hydrogels crosslinked via thiol-ene chemistry (Fig. 1C) [45]. In addition, the crosslinker composition significantly affected the shear moduli reached after curing (Fig. 1C). At higher GIA peptide contents, the modulus of the resulting hydrogels decreased, presumably due to a reduction in the efficiency of the coupling reaction with peptides such as GIA, compared to PEGDT [37]. It may also be proposed that differences in the conformation and end-to-end distance of the GIA peptide compared to PEGDT impact on the formation of loops and network defects that do not contribute to reticulation. Overall, the series of POx gels displayed stiffnesses in the range of 0.16 kPa (POx2-0) to 4.7 kPa (POx1-50). These moduli are slightly stiffer than fibrin glues used for stem cell delivery (moduli of 20 and 80 Pa for concentrations of fibrinogen of 2.5 mg/mL and 5 mg/mL, respectively). However softer synthetic POx gels were not found to be stable over prolonged incubation times.

### 3.2 Cytocompatibility of POx hydrogels

**Fig. 2:**
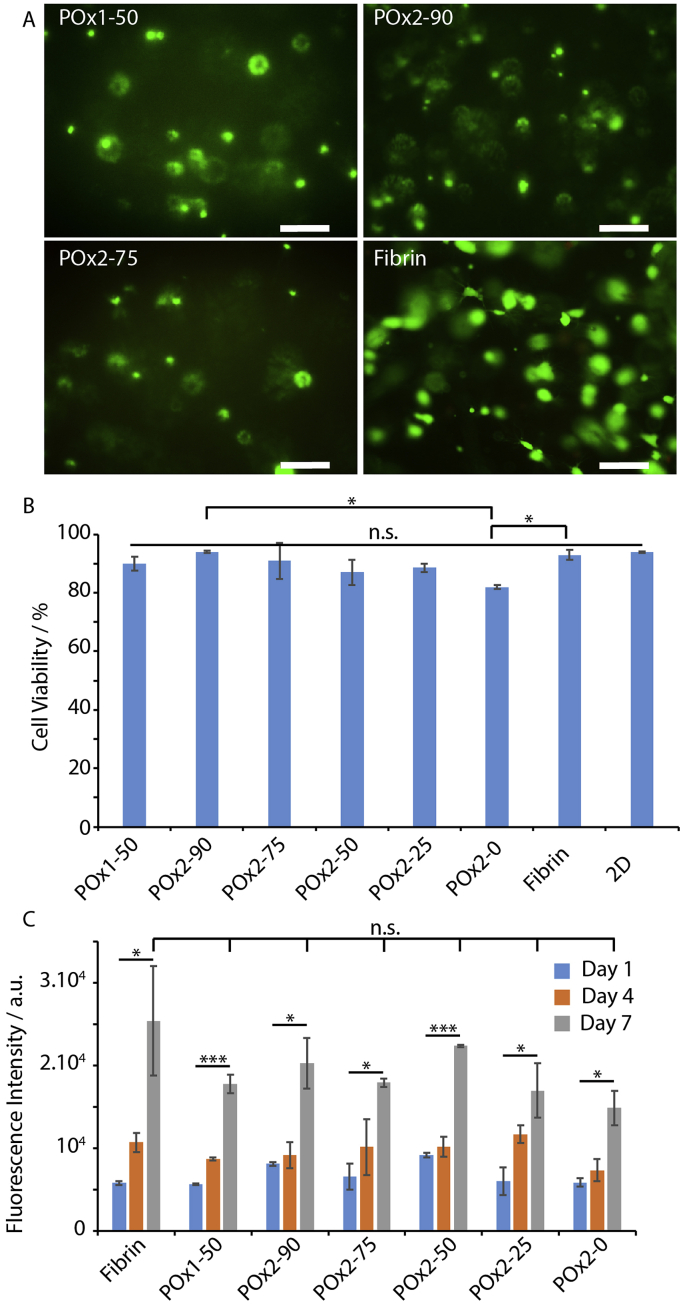
Cytocompatibility of POx hydrogels. A. Live/dead staining (green/red, respectively) of bm-MSCs encapsulated in POx hydrogels for 24 h, compared to cell encapsulated in fibrin (5 mg/mL). Scale bar, 200 μm. B. Corresponding quantified viabilities. C. Quantification of cell proliferation in POx hydrogels at days 1, 4 and 7, via CYQUANT DNA quantification assay. Results are averages ±SEM from triplicates. *p < 0.05; ***p < 0.001; n.s., not significant. (For interpretation of the references to colour in this figure legend, the reader is referred to the Web version of this article.)

The encapsulation of human bone marrow derived MSCs (bm-MSCs) in POx hydrogels was investigated next. Cells were incorporated in a POx/crosslinkers/RGD solution, mixed and quickly cured under UV photoirradiation for 120 s. This clearly led to the gelation of the cell suspensions and MSC culture medium was added to each sample, prior to their transfer to an incubator. After 24 h of culture, cell viability was quantified via live/dead assay (Fig. 2A and B). We observed no significant cytotoxicity compared to cells encapsulated in fibrin hydrogels, with cell viabilities ranging from 87 to 94%, apart from the 100% GIA-crosslinked hydrogel (POx2-0), which displayed 82% viability. To further confirm these results, we examined the proliferation of MSCs in hydrogels for prolonged culture times (up to 7 days, Fig. 2C), via DNA quantification. These results indicate that, although cell densities are comparable at early time points, cells proliferated faster in fibrin gels at later time points, although the variability observed in fibrin gels did not lead to statistical significance between the different conditions tested. We noted that live-dead staining and fluorescence images after phalloidin staining (see below) displayed higher densities in fibrin gels after prolonged culture, which is the result of the ability of MSCs to migrate within fibrin gels close to the bottom substrate.

### 3.3 Cell spreading and cell-mediated degradation in POx hydrogels

We investigated first the stability of cell-loaded hydrogels. When gels of varying compositions were left in medium without cells, they remained stable for at least 14 days. In contrast, cell-loaded hydrogels in which the GIA peptide was the sole crosslinker degraded in 3 days and cells were found spread at the bottom of the corresponding well (Supplementary Figs. S3 and S4). When PEGDT was the sole crosslinker, degradation was not apparent and cells remained suspended in the gels for at least 7 days. Intermediate formulations led to a steady increase in the degradation rate as the GIA peptide content was increased in the hydrogel formulation. These results suggested that cell-mediated degradation was able to lead to matrix remodelling and cell spreading, as observed in other thiol-ene hydrogels [46].

**Fig. 3:**
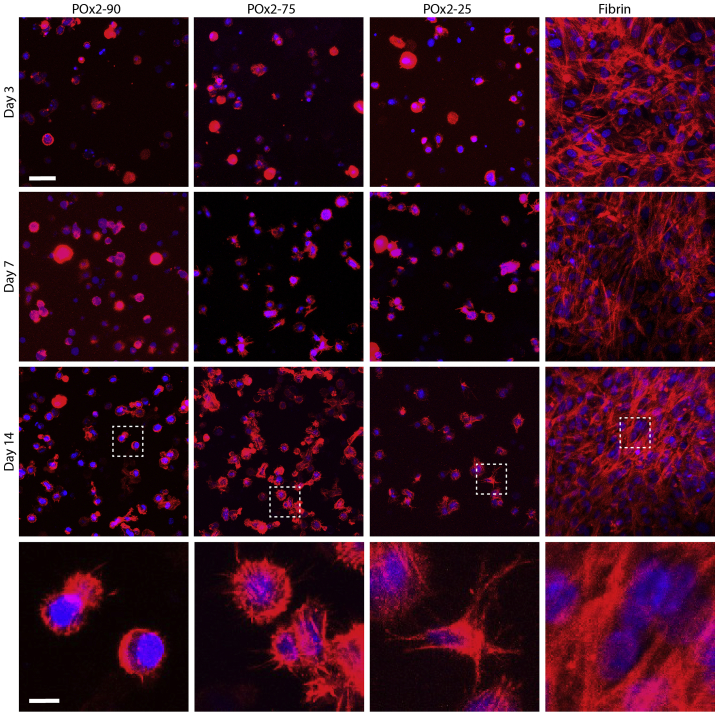
Impact of hydrogel composition on ha-MSC morphology. Cells were seeded at 100,000 cells/mL in POx2-90, POx2-75, POx2-50 and fibrin hydrogels and cultured in normal ha-MSC medium. Their morphology was observed at days 3, 7 and 14. Blue, DAPI; red, phalloidin. Scale bar, 50 μm. Bottom row: zooms of images taken at day 14. Scale bar, 10 μm. (For interpretation of the references to colour in this figure legend, the reader is referred to the Web version of this article.)

In order to study changes in cell morphology, we seeded bm-MSCs and ha-MSCs in POx hydrogels (100,000 cell/mL) and investigated their ability to remodel the matrix, protrude and elongate in these environments (Fig. 3 and Fig. S3). In fibrin gels (5 mg/mL), phalloidin stainings clearly indicate cell spreading and significant elongation, even after 3 days of culture. In POx hydrogels, spreading was far more restricted. When PEGDT was used as crosslinker or at higher POx polymer content (POx1-50), we did not observe any evidence of protrusions (Supplementary Fig. S3). When GIA was the sole crosslinker (POx2-0), we clearly observed cell-mediated gel degradation and cells appeared very spread, but all adhering to the underlying glass substrate (Supplementary Fig. S3). At intermediate contents of GIA/PEGDT, we observed increased numbers of protrusions in hydrogels crosslinked with higher contents of GIA, especially at days 7 and 14. With ha-MSCs, we observed some evidence of cell spreading in POx2-25 after 14 days of culture (Fig. 3). We noted that ha-MSCs overall displayed increased numbers of protrusions and more deformed morphologies than bm-MSCs, perhaps reflecting differences in their ability to remodel POx hydrogels or differences in expression of integrins by these cells. Overall, we found that cell spreading was significantly reduced in POx gels, compared to fibrin gels. This is in agreement with results reported for MSCs spreading in hyaluronic acid hydrogels crosslinked via thiol-ene chemistry and cell degradable peptides, although cell spreading was found to be more pronounced in these matrices [21,22]. However, our results clearly indicate that cell-degradable POx hydrogels allow to control cell protrusion and spreading, whilst enabling to retain cell encapsulation and preventing full disruption of the hydrogel constructs.

### 3.4 Encapsulation in POx hydrogels regulates the secretory phenotype of MSCs

**Fig. 4:**
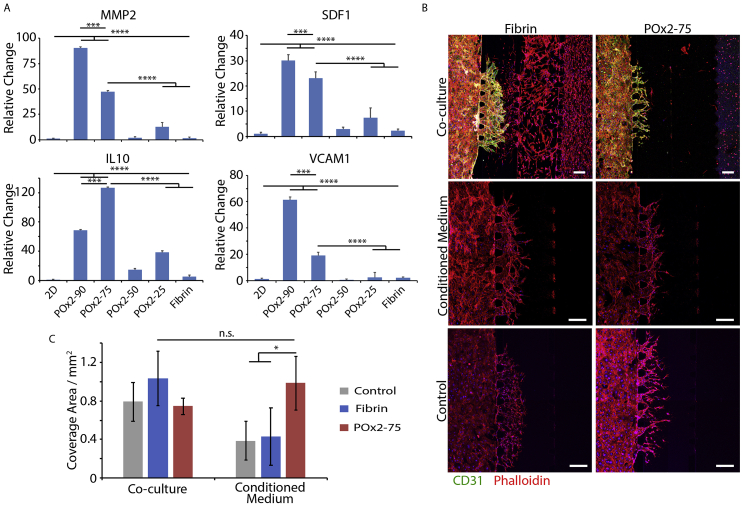
Encapsulation in POx hydrogels regulates the secretory phenotype of MSCs. A. Quantification of gene expression of key factors promoting cardiac repair by MSCs in POx hydrogels, after 3 days of culture. ***, p < 0.001; ****, p < 0.0001. B. HUVECS sprouting within the central channel of a microfluidic chip filled with fibrin gel (5 mg/mL), in the absence of VEGF but in the presence of an interstitial flow (medium level imbalance, 200 μL on the right vs. 160 μL on the left of the chip; see methods). Sprouting time: 7 days. In control and conditioned media conditions, the far right gel channel was filled with the corresponding gel (fibrin or POx2-75) but without bmMSCs and the supplemented medium was either basal EBM2 (Control) or conditioned EBM2 (by bmMSCs cultured in fibrin or POx2-75). In the co-culture conditions, the gels introduced in the far right channel (fibrin or POx2-75) contained bmMSCs at a density of 100,000 cells/mL (10 μL injected). Scale bar 100 μm. Blue, DAPI; Red, Phalloidin; Green, CD 31. C. Corresponding quantification of the area covered by angiogenic sprouts after 7 days of culture. n.s., not significant; *, p < 0.05. (For interpretation of the references to colour in this figure legend, the reader is referred to the Web version of this article.)

To characterize the secretory phenotype of MSCs cultured in POx gels, the expression level of several genes associated with improved cardiac repair was quantified by RT-PCR [8,9,28]. To do so, bm-MSCs were cultured in a range of POx gels and fibrin gels for 72 h prior to harvesting and purification of associated RNA. We focused on GIA-containing formulations to ensure that the gels would be able to degrade fully, in response to cell secretion of matrix digesting enzymes. RT-PCR clearly indicated the upregulation of MMP-2, SDF-1, IL-10 and VCAM-1 compared to cells cultured in fibrin and on 2D plastic (Fig. 4A). In contrast, the expression levels of IGF, VEGF, TGFβ1, HGF and TIMP1 were not found to be significantly altered compared to 2D plastic or fibrin (Supplementary Fig. S4; except for POx2-90 for IGF and VEGF; see Supplementary Tables S3–S11 for full statistical analysis). Paracrine secretions of MSCs delivered for cardiac repair are proposed to regulate cell proliferation in the infarcted tissue, to reduce the inflammatory response and promote angiogenesis, promoting tissue repair [2,12,47]. In this respect, POx2-90 and POx2-75 hydrogels displayed particularly striking upregulation of MMP-2, SDF-1, IL-10 and VCAM-1 compared to other groups, associated with angiogenesis in cardiac repair [8,48,49]. Therefore, these two hydrogels were selected for further testing in a functional assay (an *in vitro* angiogenesis assay based on a microfluidic chip system) to examine whether secretions from cells cultured in corresponding conditions were sufficient to induce sprouting of endothelial cells.

The microfluidic model used to study angiogenesis were based on systems previously developed for the control of vascular networks formation in multi-channel microfluidic chips [50,51]. In this system, microfluidic chips were designed to display 4 parallel channels separated by posts (700 μm in length and 100 μm wide), therefore enabling the retention of hydrogel solutions in selected channels prior to gelation. The left and center-right channels were selected as media channels, whereas the center-left channel was selected as fibrin gel (5 mg/mL) channel within which human umbilical vein endothelial cells (HUVECs) were allowed to invade from the left channel (after seeding in this channel at 2.0 M cells/mL). In the right channel, MSC-loaded hydrogels (fibrin 5 mg/mL or POx2-75 gels with 1 million cells/mL) were introduced, to act as growth factor producing compartment. Alternatively, conditioned culture medium was directly introduced in the center right channel. An interstitial flow was established against the HUVECs sprouting, via an imbalance in the medium level across the center right-left channels 200 μL in the center right and 160 μL in the left channel), as this was found to stimulate the progression of angiogenic sprouts [52]. It should be noted that no additional growth factors (e.g. VEGF) were supplemented to the basal culture medium (EBM-2) in any of the conditions investigated. In such conditions, in the absence of additional pro-angiogenic factors, even with interstitial flow, sprouting was severely limited.

After 7 days of co-culture, HUVEC sprouting into the center-left fibrin channel is clearly observed as a result of MSC secretions (Fig. 4B and C). This is particularly pronounced in the case of MSC co-culture in fibrin gel (in the right channel), however very significant invasion of MSCs within the center-right and even the center-left channel are likely to result in higher local concentrations of proangiogenic factors and associated gradients. In contrast, MSCs seeded in POx2-75 remained encapsulated in this gel throughout the experiment, yet established sufficiently high concentrations of proangiogenic factors to result in significant sprouting of HUVECs within the center-left channel. Confocal imaging also clearly indicated the formation of lumenated networks in the co-cultured groups, indicating the formation of relatively mature vessels and the pro-angiogenic potential of MSC-loaded hydrogels (Supplementary Fig. S5, although apico-basal polarity was not characterised). In contrast, control groups (no MSCs) displayed sprouting but did not lead to the formation of lumenated structures (Supplementary Fig. S6). This is consistent with observations made in a very similar model that a low interstitial flow alone (applied via a simple imbalance in media levels across the central channel) was able to induce some sprouting, even in the absence of any supplemented growth factor [53]. However, when angiogenesis experiments were repeated with conditioned media, comparable sprouting lengths were observed when HUVECs were stimulated with conditioned medium, obtained from MSCs cultured in fibrin or POx2-75 hydrogels (Fig. 4B and C). The comparable VEGF expression that was observed for MSCs cultured in these gels (Supplementary Fig. S4) is in good agreement with this observation. In contrast, the overexpression of MMP-2, SDF-1, IL-10 and VCAM-1 does not correlate with the promotion of angiogenesis in this model and further experiments would be required to establish their role in establishing such phenotype. Hence, overall, our results imply that MSCs encapsulated in POx2-75 hydrogels display a proangiogenic secretory phenotype and overexpress a number of growth factors associated with effective cardiac repair.

### 3.5 POx hydrogel tissue adhesion and mechanical stability

**Fig. 5:**
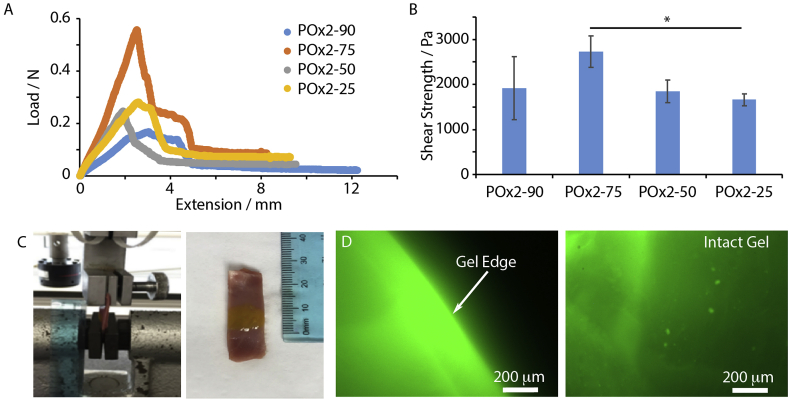
Characterization of hydrogel adhesion to the epicardium and mechanical stability. (A) and (B). Shear strength of different POx hydrogels cured in contact with the epicardium. The contact length in the samples was 1 cm *p < 0.05. No significant difference was observed between other groups. (C). FITC labelled POx2-75 was cured on epicardium tissue prior to direct stretch of the tissue. (D). Fluorescence image of the POx2-75 gel, after stretching (50% strain). Scale bar 200 μm.

As we aimed to deliver MSCs to infarcted cardiac tissues using an epicardial placement approach, POx hydrogels were expected to provide strong adhesion to the surface of the epicardium and mechanical integrity during cardiac beating, therefore enabling the retention of cells, as well as enhancing the secretion of growth factors. Gel adhesion was characterized to porcine cardiac tissues. POx hydrogels, including POx2-90, POx2-75, POx2-50 and POx2-25 were cured via photoirradiation and thiol-ene chemistry, directly in contact with the epicardium of porcine cardiac tissues, sandwiched in between a PMMA slide and glass slide, for mounting in a tensile tester. Shear lap testing was carried out, to measure the associated shear stress and quantify hydrogel adhesion to the epicardium (Fig. 5A and B). We observed relatively high bonding, in particular in the case of POx-2 75, with a shear stress of 2780 ± 350 Pa.

We next studied the mechanical stability of gels adhered to the epicardium, during uniaxial stretch of the samples (50%). The tissue sample (porcine cardiac tissue coated with POx2-75 gel labelled with 5 mol% of FITC-RGE peptide to enable fluorescence imaging of the resulting interfaces after stretching) was mounted in the tensile tester (Fig. 5C). After 50% strain, an intact gel layer at the surface of the epicardium (Fig. 5D). Therefore, we concluded that POx2-75 hydrogels could bear the stretch and extension resulting from cardiac beating. This POx gel therefore appeared to display the set of biochemical, bioactive and mechanical properties suitable for the encapsulation of MSCs and epicardial placement in an *in vivo* model.

### 3.6 ha-MSCs loaded in POx gels enhance cardiac function in a MI rat model

Prior to animal experiments, we first set out to confirm the suitability of the photocuring protocols for the encapsulation of ha-MSCs in POx hydrogels for epicardial placement. We first confirmed that cell viability remained high on a wide range of cell loading densities (0.5–2 million cells/mL, Supplementary Figs. S7A and B). After 1–3 days of culture viabilities were comparable at all densities. We also confirmed via photo-rheology that the kinetics of photocuring and the mechanical properties of the resulting hydrogels were not significantly impacted by a high cell density typical of epicardial placement experiments (2 million cells/mL, Supplementary Figs. S7C and D). After 120 s photoirradiation, we indeed observed comparable mechanical properties for hydrogels with and without ha-MSCs. However, the rate of strengthening of the shear storage modulus during gelation was slightly reduced in the presence of ha-MSCs. Therefore, our results confirm that 120 s photo-irradiation should be sufficient for setting of cell-loaded POx hydrogels for epicardial placement, but that reduction of this time would be detrimental to the mechanical properties of the resulting hydrogel.

In general, POX gels demonstrated slower degradation compared to fibrin gels. On day 14, multilayer of gels and round cells could be detected in different conditions of POX gels, however cells were all spreading in a thin layer of remaining fibrin gels. Thus it demonstrated that POX gels degraded slower than fibrin gel, which would enhance cell retention when applied on *in vivo* models for long term observation.

**Fig. 6:**
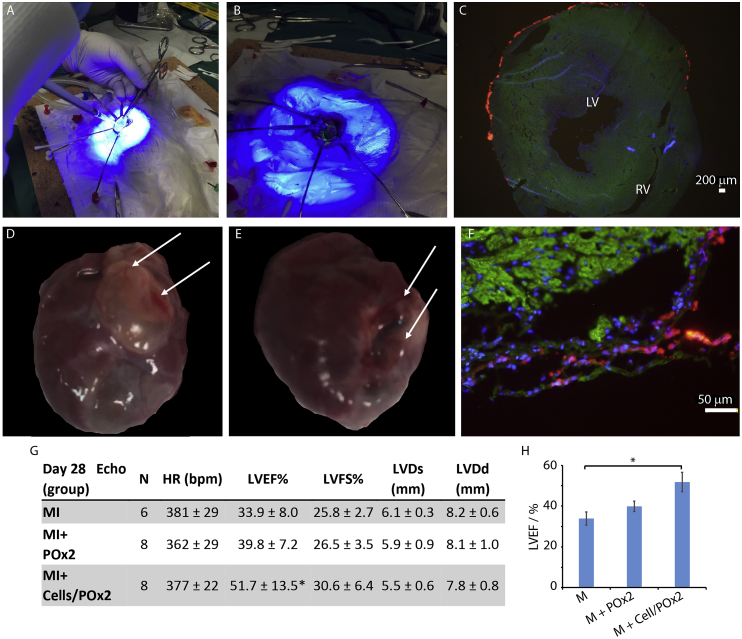
Epicardial placement of haMSC-loaded POx2-75 hydrogels in a MI rat model. A and B. Acute MI rat model creation and hydrogel photo-curing during (A) and just after epicardial placement (B). C. Retention of ha-MSCs loaded in POx2-75 hydrogels at the surface of the epicardium, immediately after UV photo-curing. Blue, DAPI; green, α-sarcomeric actin; red, CM-Dil labelled ha-MSCs; scale bar, 200 μm. LV, left ventricle; RV, right ventricle. D and E. Macroscopic view of POx hydrogels (MI + Gel and MI + Gel + Cell samples) adhered to the heart surface 28 days post-surgery. Arrows point to the gel layer). F. CM-Dil labelled ha-MSCs retained at the surface of the epicardium 28 days post-surgery. Green, α-SA; blue, DAPI; red, CM-Dil labelled ha-MSCs; scale bar, 50 μm. G and H. Quantification of cardiac function on day 28 post-surgery (bpm, beat per minute; LVEF, left ventricular ejection fraction; LVFS, left ventricular fractional shortening; LVDs, left ventricular end-systolic dimension; LVDd, left ventricular end-diastolic dimension). Results are reported as averages ± standard errors. *p < 0.05 vs MI group. (For interpretation of the references to colour in this figure legend, the reader is referred to the Web version of this article.)

Following the optimisation of POx hydrogel retention on sacrificed rat cardiac tissues (e.g. two stage photo-curing to limit the fluidity of the POx/cell pre-curing mixtures and improve retention, see methods section 2.8), we next investigated the impact of POx2-75 and cell-loaded POx2-75 hydrogels on cardiac function on a rat MI model. Left thoracotomy was performed on rats under anesthesia and the left coronary artery was ligated to induce acute MI. The macroscopic view of an open chest of the surgery area is shown in Fig. 6A/B. To ensure full retention of the hydrogel at the surface of the epicardium, a sterile cylinder was used to localize the POx/cell mixture during photo-irradiation. This resulted in the formation of a cell-loaded hydrogel with approximately 1 cm cross-section and 2.5 mm in thickness. To examine gel retention post-curing, an FITC-labelled RGE peptide was introduced in the gelation system in some of our preliminary experiments and allowed to confirm the retention of the gel at the surface of the epicardium.

To investigate gel/cell retention during epicardial placement, CM-Dil labelled ha-MSCs were placed on the epicardium of intact rats using our POx hydrogels (as a cell/gel suspension) and the hydrogel was cured as described in previous sections. Images of histological sections clearly indicate the retention of a hydrogel-cell layer on the anterior wall of the left ventricle of the infarcted heart (Fig. 6C).

Finally, for the examination of the impact of MSC delivery to the epicardium via POx hydrogel placement, acute MI was induced in male Lewis rats. Three experimental groups were selected: MI only (MI, N = 10), MI induction followed by POx hydrogel placement (MI + Gel, N = 10), MI induction followed by ha-MSC-loaded POx hydrogel (MI + Gel + Cell, N = 10). 2 rats from the MI group did not survive the MI induction and surgery and the rest of 28 rats were kept and observed for 4 weeks post surgeries.

The echocardiography data at day 28 indicated a recovery of cardiac function in MI + Gel and MI + Gel + Cell groups, compared to the MI group. Specifically, the left ventricular ejection fraction (LVEF %) was increased by over 15%, in the MI + Gel + Cell, group compared to the MI group (Fig. 6 G and H). Such increase compares favourably to the LVEF % increase reported following epicardial placement with other biomaterials (e.g. LVEF % increase of 9.7% was reported for a fibrin glue [28], whereas PuraMatrix delivered cells allowed an increase in LVEF % of +8.5% [9]). With histologiocal donor cell trafficking using CM-DIi-labelled MSCs, we observed that ha-MSCs remained present at the surface of the epicardium even after 28 days in the hosts (Fig. 6F and Supplementary Fig. S8). To quantify the survival of donor cells, the human specific gene ALU was quantified by PCR and its expression level was compared to a standard curve generated from known ha-MSC densities (Supplementary Fig. S9). After 28 days implantation, cell survival rate was 3.5 ± 1.7%, comparable to survival reported for transendocardial injection of allogenic porcine bone marrow MSCs to pig heart (6% after 10 days injection) [54]. Such high survival rates, compared to other strategies proposed for the xenogeneic transplantation of human bone marrow derived MSCs to immunodeficient murine heart (<0.44% of donor MSCs survived after 4 days engraftment) [55] or allogenic transplantation of bone marrow MSCs to infarcted rat hearts by IM injection (1% donor cells 24 h after transplantation) [56] are proposed to be an important factor to the performance of MSC-loaded POx gels to promote the recovery of cardiac functions.

### 3.7 haMSC-loaded POx hydrogels reduce interstitial collagen deposition and infarct size and increased wall thickness in the MI rat model

**Fig. 7:**
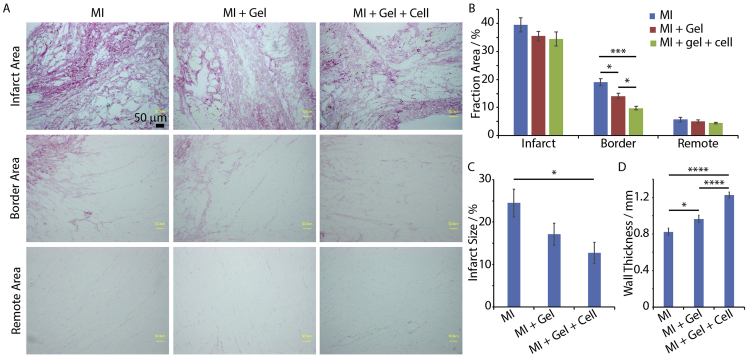
ha-MSC loaded POx2-75 hydrogels reduced interstitial collagen deposition and infarct size and increased wall thickness in MI rat models. A. Picrosirius Red Staining showed collagen deposition in infarct, border and remote areas of infarcted hearts. Scale bar 200 μm. B. Quantification of collagen deposition. MI group (N = 3), MI + Gel group (N = 4) and MI + Gel + Cell group (N = 4). C and D. Quantification of infarct size and wall thickness. MI group (N = 3), MI + Gel group (N = 4) and MI + Gel + Cell group (N = 4). *p < 0.05; ****p < 0.0001. (For interpretation of the references to colour in this figure legend, the reader is referred to the Web version of this article.)

Collagen deposition, scar size and left ventricle wall thickness were evaluated by Picrosirius Red staining (Fig. 7A and B and Supplementary Fig. S10). Different areas of the heart samples, including the infarct area, border area and remote area, were examined. In the border area, we observed reduced collagen deposition in the MI + Gel + Cell (N = 4) group compared to the MI and MI + Gel (N = 4) groups. This was also associated with reduced infarct size and increased wall thickness. Therefore, our results indicate reduced fibrotic response of the cardiac tissue post-surgery in the group treated with MSC-loaded POx hydrogels.

### 3.8 ha-MSCs loaded POx hydrogels enhanced neovascularization in border areas of infarcted hearts and enhanced gene expression of reparative factors

**Fig. 8:**
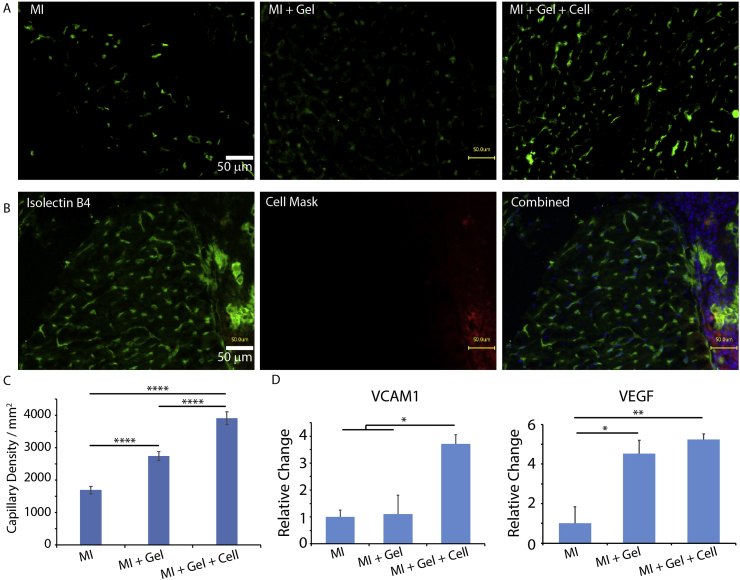
ha-MSC loaded POx2-75 hydrogels enhanced neovascularization in the border area of MI hearts and enhanced gene expression of cardiac regenerative factors. A. Isolectin B4 staining demonstrated increased capillary density in the border area of MI + Gel and MI + Gel + Cell treatment groups compared to the MI group; scale bar, 50 μm. B. Capillaries formed inside of the POx hydrogel layer. Red, CM-Dil labelled ha-MSCS; blue, DAPI; green, Isolectin B4; scale bar, 50 μm. C. Corresponding quantification of capillary densities. MI group (N = 3), MI + Gel group (N = 4) and MI + Gel + Cell group (N = 4). D. Quantification of VCAM-1 and VEGF gene expression in different MI groups (N = 3 for each group). *p < 0.05; **P < 0.01; ****p < 0.0001. (For interpretation of the references to colour in this figure legend, the reader is referred to the Web version of this article.)

To better understand the regenerative impact of POx and ha-MSC loaded POx hydrogels, micro-capillaries in the border area of cardiac tissues were characterised via isolectin B4 staining (Fig. 8A–C). Since the border area is the peri-infarcted region and neovascularization is crucial for tissue regeneration during cardiac repair after MI, we specifically characterised neovascularization in the border area, 28 days post-surgery. We observed a clear enhancement of capillary formation in the border area of infarcted hearts in MI + Gel and MI + Gel + Cell groups compared to the MI group. In addition, newly formed capillaries were not only detected in the cardiac tissue, but also at the interface between the cardiac tissue and POx hydrogel, which may contribute to the integration and retention of the gel to the surface of the epicardium (Fig. 8C). In addition, analysis of gene expression of factors associated with improved cardiac repair indicated that VCAM-1 and VEGF were significantly upregulated in the MI + Gel + Cell group, compared with the MI group (Fig. 8D and Supplementary Fig. S11). Therefore, our results indicate that epicardial placement of ha-MSC-loaded POx hydrogels results in a significant improvement of vascularisation in a MI rat model.

## 4 Discussion

Regulating the degradability of synthetic hydrogels via crosslinking with cell-degradable peptides (e.g. cleaved by MMPs) was previously shown to allow the control of cell spreading in 3D hydrogels [17,22,57]. However, cell spreading in such matrices displays significant differences compared to the rapid spreading observed in fibrin or collagen hydrogels. Indeed, we observed that MSC protrusion was very severely reduced in POx hydrogels compared to the rapid spreading (within 1 day, results not shown) observed in fibrin hydrogels (Fig. 3). Although we observed some level of protrusion at day 3 in the most degradable POx hydrogels, cell elongation was not observed until day 14. This is in general agreement with cell spreading (fibroblasts and MSCs) reported in other degradable synthetic hydrogels generated via thiol-ene coupling, although the chemistry of the backbone and that of the degradable peptide crosslinker significantly modulate such phenotype [17,22]. In contrast, in the absence of degradable crosslinks, cells are typically unable to spread in 3D matrices, although they can remain viable and deposit pericellular matrix, regulating their phenotype [22,46]. Spreading in 3D POx hydrogels, even in the presence of degradable peptides, was not observed previously (although 3D encapsulation of fibroblasts in POx hydrogels was previously explored by Dargaville and coworkers [35,36]) and our results confirm that, similarly to hyaluronan or PEG-based hydrogels [17,22,57]. It is possible to modulate MSC spreading using such backbones. However, we note that protrusions remain more limited in POx hydrogels compared to charged hyaluronan hydrogels or PEG hydrogels crosslinked via faster degradable peptides. We also observed that, once cells managed to spread further in POx hydrogels, they were rapidly found to migrate to the underlying substrate where they can spread to a greater extent, in a quasi 2D environment (see Supplementary Fig. S3). This suggests that matrix remodelling in POx hydrogels is incomplete (at least *in vitro*) and that cells are not able to deposit a new matrix as GIA crosslinks are degraded.

In addition to the controlled regulation of cell encapsulation and cell-mediated degradation, hydrogels used for stem cell delivery are required to promote tissue adhesion, in order to sustain high cell retention and preserve mechanical integrity of the regenerating tissue. This is particularly challenging in the context of cardiac repair, as cyclic cardiac beating induces considerable mechanical stress at the tissue-hydrogel interface. Echocardiography measurements indicate that the left ventricular percent fractional shortening normally falls within 30–50% [58]. This correspond to changes in circumference (approximated based on a spherical geometry) in the range of 10–15%. In order to resist the cyclic stresses generated at the tissue-gel interface, as well as within the hydrogel layer itself, scaffolds used for epicardial placement are required to display engineered mechanical and adhesive properties. For example, hyaluronic acid hydrogels required fixation to the infarcted tissue via a fibrin glue [10]. Similarly, cardiac patches usually required suture or the use of fibrin glue to improve adhesion [59,60]. In this respect, the direct bonding of hydrogels to the surface of tissues, via thiol-ene coupling is a clear advantage. In the case of the POx formulations reported in this study, we propose that residual alkene moieties present in the formulation used enable bonding to thiol residues at the surface of the epicardium (whether by cell membrane or the extracellular matrix). The excellent mechanical stability observed for POx hydrogels up to 50% strain and the absence of apparent fracture within the hydrogel (Fig. 5) indicated that thiol-ene based adhesion and shear mechanical properties should allow to sustain the contractile and diastolic response of the beating heart following epicardial placement. In addition, the neutral character of POx hydrogels also limits local changes in the pH of the epicardium microenvironment, a factor that may otherwise reduce cell viability.

Donor cell retention is a key factor to the success of cell transplantation therapies for cardiac repair. In this respect, the *in situ* gelation of cell-loaded POx hydrogels helped to achieve high initial cell retentions. Even 28 days post-surgery, significant levels of retention of ha-MSCs were observed within the POx hydrogel, at the surface of the epicardium (Fig. 6). The slow controlled degradation rate of the POx hydrogel may be a factor benefiting such cell retention for long-term implantation. Overall, after 28 days, the donor ha-MSC survival rate was found to be 3.5 ± 1.7% (Supplementary Fig. S9). This compares well to other stem cell delivery and epicardial placement strategies. Hence, the trans-endocardial injection of allogenic porcine bone marrow MSCs to pig heart is associated with a cell survival rate of 6% after 10 days, but expected to decrease as a function of time [54]. In contrast, xenogeneic transplantation of human bone marrow derived MSCs to immunodeficient murine hearts was associated with a survival rate of donor MSCs of 0.44%, 4 days post-engraftment [55]. Similarly, the allogenic transplantation of bone marrow MSCs to infarcted rat hearts by IM injection resulted in 1% of donor cells detection 24 h post-transplantation [56]. Therefore, our results indicate an excellent level of cell retention of survival 28 days post-surgery in our MI rat model, a factor that is proposed to improve the recovery of cardiac function.

Epicardial placement of Pox-MSC complex displayed an increased LVEF% compared to the MI group (Fig. 6). It is likely that this improvement was associated with the secretory phenotype of implanted cells [2,47,61]. *In vitro*, we observed a clear upregulation of MMP-2, SDF-1, IL-10, VCAM-1 and VEGF in POX gels compared to cells cultured in fibrin or on 2D plastic (Fig. 4). Although the mechanism controlling the modulation of cytokine expression in 3D hydrogels remains unknown (including in POx hydrogels), it could be speculated that the limited cell protrusion observed in POx hydrogels may result in the upregulation of factors associated with matrix remodelling. MMP-2 is typically associated with matrix degradation and the modulation of migratory phenotypes [62]. SDF-1 was reported to play a critical role in the regulation of MSC survival, migration and cytokine secretion, and to enhance the secretion of VEGF and bFGF via CXCR4 [63]. IL-10 is an anti-inflammatory cytokine that helps cell retention and was reported to improve cardiac function [64] and to play a significant role in modulating the expression of MMPs and TIMP-1, suppressing the synthesis of pro-inflammatory cytokines in ischemic area of the heart [65]. VCAM-1 regulates cell adhesion and plays a critical role in immunosuppression of MSCs by increasing adhesion between MSCs and T cells [66].VCAM-1 was proposed to be responsible for the migration of endothelial cells and the prevention of cardiomyocyte apoptosis in MI mice models [67]. VEGFs regulate angiogenesis and vasculogenesis and was found to be a key trophic factor improving MSC survival [48] and correlating with cardiac function recovery [68]. Therefore, our *in vitro* and *in vivo* results (Fig. 4, Fig. 8) indicate the upregulation of key cytokines and growth factors associated with cardiac repair.

Consistent with such observations, neovascularization was found to be enhanced in ha-MSC loaded POx hydrogels (Fig. 8). Newly formed capillaries were detected in the per-infarct ischemic area. Neovascular formation was also observed between ha-MSC loaded POx gels and the epicardium (Fig. 8B). Such connected capillaries may play an important role in the regeneration process. Indeed, such vascularisation was proposed to promote survival of nearby cardiomyocytes and inhibit apoptosis [69]. This inspired the strategy of delivering micro-engineered blood vessels in cardiac patches for treatment of MI [70].

Correlated with the improved neovascularization of the cardiac tissue in the MI + Gel + Cell group, we observed a significantly decrease in collagen fraction in the border area of the ischaemic epicardium, compared to the MI group (Fig. 7). In addition, the MI + Gel + Cell group was associated with a significant decrease in infarct size and increased wall thickness, compared to the MI group (Fig. 7). Following MI, two types of fibrotic responses were reported to take place, including replacement fibrosis (collagen deposition associated with scar formation in the infarcted area) and reactive fibrosis (interstitial fibrosis in the border area and remotely in the uninjured myocardium) [71]. Although replacement fibrosis plays an important role in preventing heart failure, an exaggerated fibrotic response and interstitial fibrosis can cause dysfunction of the left ventricle and eventually lead to heart failure [72]. Therefore regulation of the process of reactive fibrosis will contribute to prevent the adverse remodelling of the non-infarcted left ventricular wall [73].

As an additional mechanism of cardiac function improvement by Pox-MSC complex, the mechanical properties of the POx hydrogel (shear modulus of 1.8 kPa, Fig. 1) may act as a soft scaffold supporting the injured heart and restrain its dilation, although this effect will be limited considering the mechanical properties typically associated with the epicardium (the native myocardium is reported to display 10–20 kPa moduli at the beginning of diastole and 200–500 kPa at the end of diastole) [74,75]. Hence, it was reported that engineered acellular collagen patches (2–10 kPa elastic moduli) enhanced cardiac function through mechanical support [76]. Although such mechanical protection may play a role in the minor improvement to cardiac function observed, it is also likely that the placement of an empty POx hydrogel may have resulted in a mild foreign body response, contributing to the innate inflammatory response post-MI and associated limited cardiac repair [77,78]. Modulation of the inflammatory cascade is indeed proposed to be an important therapeutic strategy for cardiac repair [77,78]. Indeed, it was reported that POx materials demonstrated some immunomodulatory properties, activating macrophages and inducing the release of pro-inflammatory cytokines [79]. However, further investigation is required in order to fully establish whether POx hydrogels alone are able to improve cardiac function via such mechanism and whether this could be improved. Finally, we note that human amnion-derived MSCs were not found to cause significant immunological response when transplanted into the heart of rodents, potentially due to their immunomodurative secretion ability [7], although their impact in the particular context of this study was not evaluated and deserves further study.

## 5 Conclusion

In conclusion, the design of degradable POx hydrogels is found to be an attractive strategy for the epicardial placement of MSCs. Importantly, we report that the mechanical and cell-degradable properties of POx hydrogels can be tuned to regulate the secretory phenotype of MSCs, in particular stimulating the secretion of cytokines and growth factors promoting vascularisation. This was validated *in vitro* at the gene expression level, but also using a novel microfluidic angiogenesis platform, highlighting the role that advanced *in vitro* models will increasingly play in the design of novel regenerative medicine strategies. Indeed, advanced *in vitro* models and organ-on-chip systems are attractive stepping boards to fine tune the design of novel biomaterials, prior to evaluation in animal models. The pro-angiogenic phenotype observed *in vitro* was indeed confirmed in a MI rat model, with clear neovascularization observed in the cardiac tissue and at the epicardium-hydrogel interface. This was associated with increased secretory gene expression and a reduction of collagen deposition and fibrosis. Overall, ha-MSC loaded POx hydrogels resulted in a retention of MSCs at the epicardium and the recovery of cardiac functions following myocardial infarction. Therefore, we demonstrated the feasibility of stem cell delivery within POx hydrogels via a simple method. Further design of POx materials will include the elucidation of the mechanism via which MSC secretory phenotype is regulated. In particular, how the combination of biochemical, mechanical and degradative properties of the matrix control gene expression and protein secretion.

## Credit author statement

**Yaqi You:** Methodology, Writing- Original draft preparation. **Kazuya Kobayashi:** Methodology, Supervision. **Burcu Colak:** Methodology. **Piaopiao Luo:** Methodology. **Edward Cozen:** Methodology. **Laura Fields:** Methodology. **Ken Suzuki:** Conceptualisation, Supervision, Writing- Review and editing. **Julien Gautrot:** Conceptualisation, Supervision, Writing- Original draft preparation.

## Data availability

All data analyzed during this study are included in this published article (and its supplementary information file). Other raw data required to reproduce these findings are available from the corresponding author on request.

## Declaration of competing interest

The authors declare that they have no known competing financial interests or personal relationships that could have appeared to influence the work reported in this paper.
