# Supplementary material for: Engineered cell-degradable poly(2-alkyl-2-oxazoline) hydrogel for epicardial placement of mesenchymal stem cells for myocardial repair
Source: Biomaterials. 2021 Feb;269:120356. doi: 10.1016/j.biomaterials.2020.120356 (PMC7884911; doi:10.1016/j.biomaterials.2020.120356)
Supplement: Multimedia component 1 [file mmc1.pdf]

Engineered Cell-Degradable Poly(2-alkyl-2-oxazoline) Hydrogel for Epicardial Placement of Mesenchymal Stem Cells for Myocardial Repair

Supplementary Information

Yaqi You[a,b,c], Kazuya Kobayashi[c], Burcu Colak[a,b], Piaopiao Luo[a,b], Edward Cozen[a,b], Laura Fields[c], Ken Suzuki[a,c] and Julien Gautrot[a,b]**

[a] Institute of Bioengineering and [b] School of Engineering and Materials Science, Queen Mary, University of London, Mile End Road, London, E1 4NS, UK.

[c] William Harvey Research Institute, Barts and the London School of Medicine and Dentistry, Queen Mary University of London, UK.

* To whom correspondence should be addressed. E-mail: j.gautrot@qmul.ac.uk and ken.suzuki@qmul.ac.uk

Supplementary Table S1. Human primers sequences corresponding to genes associated with cardiac function.

Gene	*Forward*	*Reverse*	*Comment*
B-2M	GAGGCTATCCAGCGTACTCCA	CGGCAGGCATACTCATCTTTT	Housekeeping gene
MMP-2	CTCTCCACTGCCTTCGATACA	GGAGGAGCCACTCTCTGGAATCT	Migration
SDF-1 (CXCL12)	CTTGTAATCCGAATCTCTTTTTGCTTT	GCCCAAGGGAGTGTCAGGTAG	Paracrine function
VCAM-1	TACTGCTCATCATTCCTTGAGAAAAAC	GCTCACAGCAAGGGACATAGA	Cell adhesion
IGF	ACCACCTTTCAACTTTTTATCACTCAC	CAACAAAACAATGGAGCCTTCTAAC	Paracrine function
HGF	CTTGAGTGATTTTTATCAAGGCCAAGT	CCCTATATTCTGTGGACTAAGCTCTCC	Paracrine function
VEGF-α	CGGCGAAGAGAAGAGACACATT	CTAGTGGTTTCAATGGTGTGAGGAC	Angiogenesis
Il-10	ACGCTTTCTAGCTGTTGAGCTGTTTT	GGCTCCCTGGTTTCTCTTCCT	Anti-inflammatory
TGF-β	CCAAAGTGAAGTTGTTTGATATGGTCT	CCAACACAAGGGCTGTTTACTATTATG	Angiogenesis
TIMP-1	ACTGATCGTGGGTGGATGAGTAAT	CTAGAGGATAAATGTCCACGCTAGGG	Migration

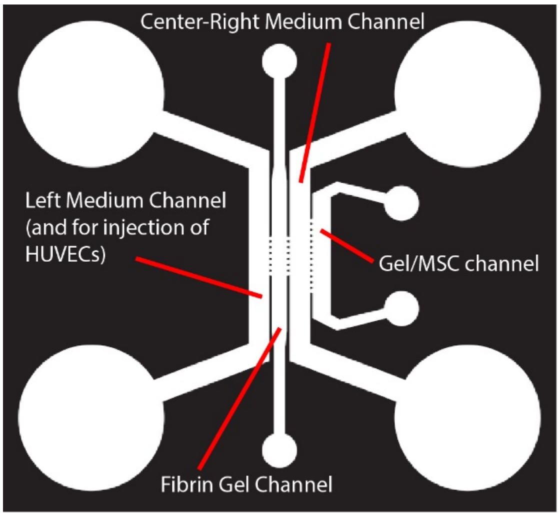

Supplementary Figure S1. Pattern of microfluidic chips used for the co-culture of HUVECs and bmMSCs. The microfluidic chips consist in 4 parallel channels (700 µm in width and 100 µm in height for each channel) separated by posts defining 100 µm gaps allowing the flow of nutrients and molecules across the different compartments.

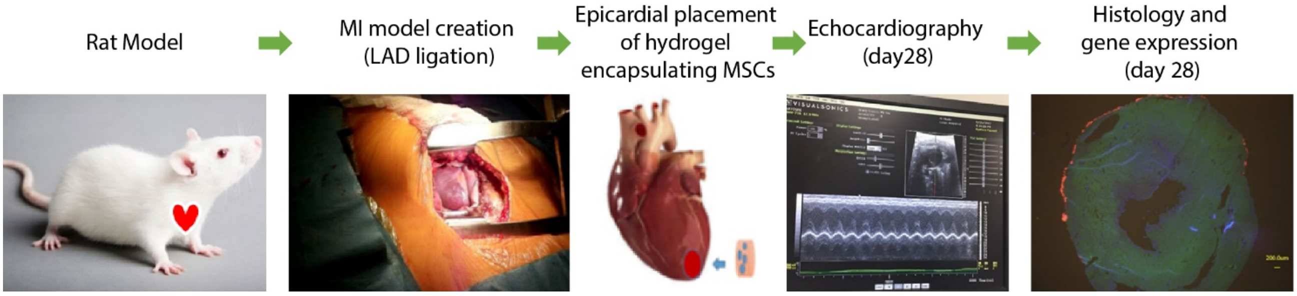

Supplementary Figure S2. Schematic representation of key steps of the *in vivo* study. 30 male Lewis rats were divided into three groups: 1. myocardial infarction induction only (MI, N=10); 2. MI induction followed by POx gels placement (MI+Gel, N=10); 3. MI induction followed by POx gel encapsulating ha-MSCs (MI+Gel+MSC, N=10). The acute MI model was created by ligation of the left anterior descending artery (LAD). For the MI+Gel group and MI+Gel+MSC group, either gel or gel plus cell mixtures were cured on the epicardium immediately after the creation of the MI model. The chest and skin were then carefully closed without disturbing the gel-cell layer and sutured. All rats were kept and observed for 4 weeks post-surgery. Transthoracic echocardiography was performed on rat models at day 28 post-surgery. Histological studies were carried out afterwards**.**

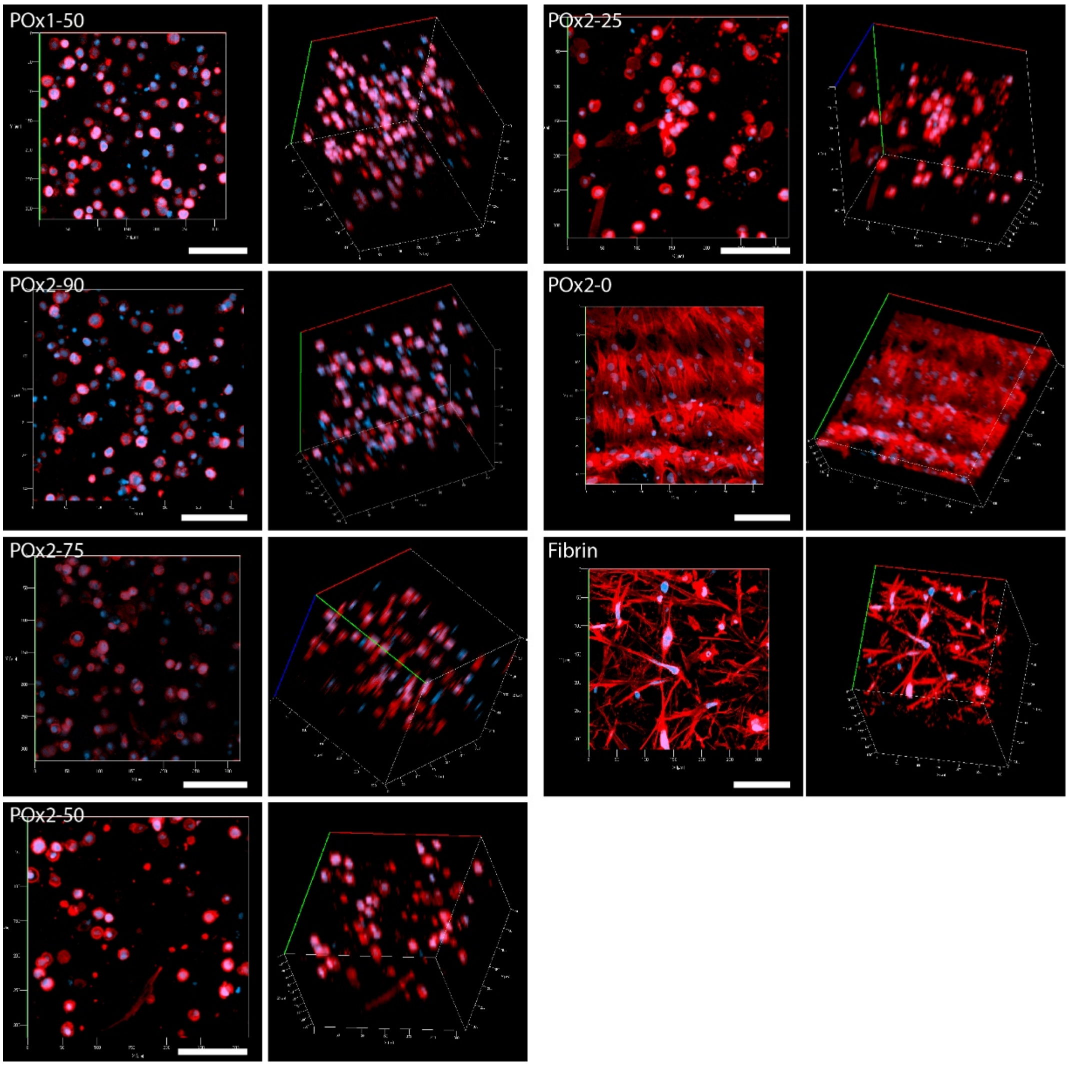

Supplementary Figure S3. Morphology of bmMSCs after culturing in different gels for 3 days. Blue, DAPI; Red, Phalloidin. Scale bars, 100 μm. Top view image dimension is 316×316 μm. Representative images selected from three different independent repeats.

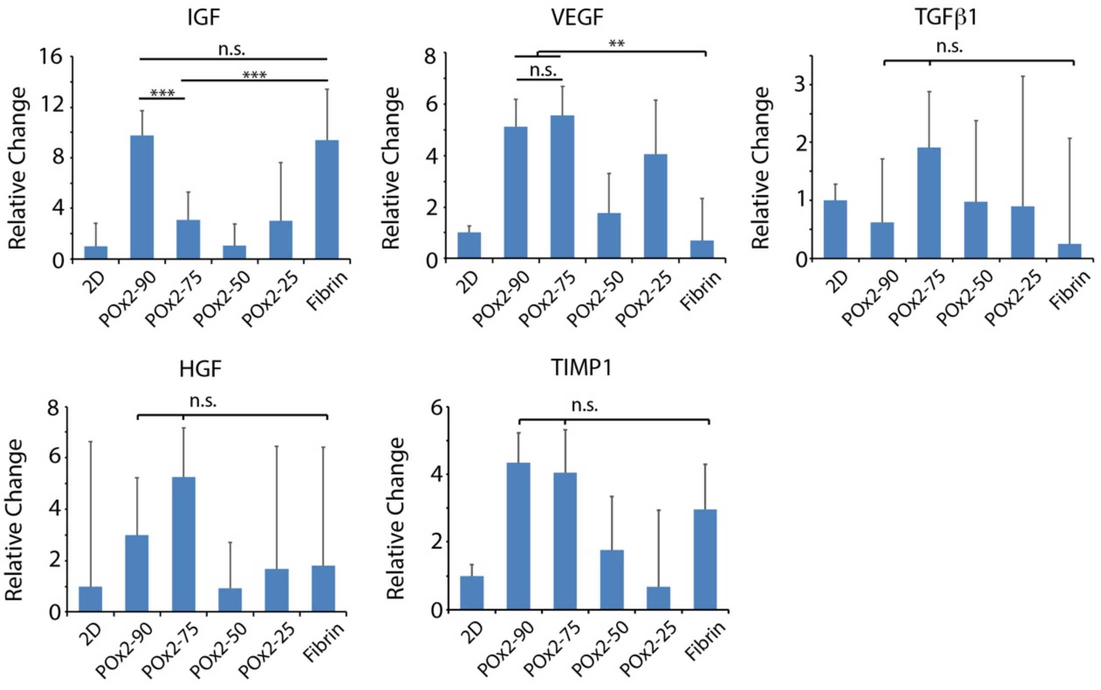

Supplementary Figure S4. Quantification of gene expression of factors associated with the secretory phenotype of MSCs promoting cardiac repair. The impact of matrix encapsulation is investigated, after culture for 3 days. n.s., not significant; ** P< 0.01; ***P<0.001. All data were normalized to β-2m housekeeping gene expression. Relative quantification of each gene was determined using the 2-ΔΔCT method. Data were analysed by GraphPad Prism 7.1. Large error bars for TIMP-1, IGF, TGF-β1 and HGF were due to the overall low expression of these genes, n=3 (three biological replicates for each group).

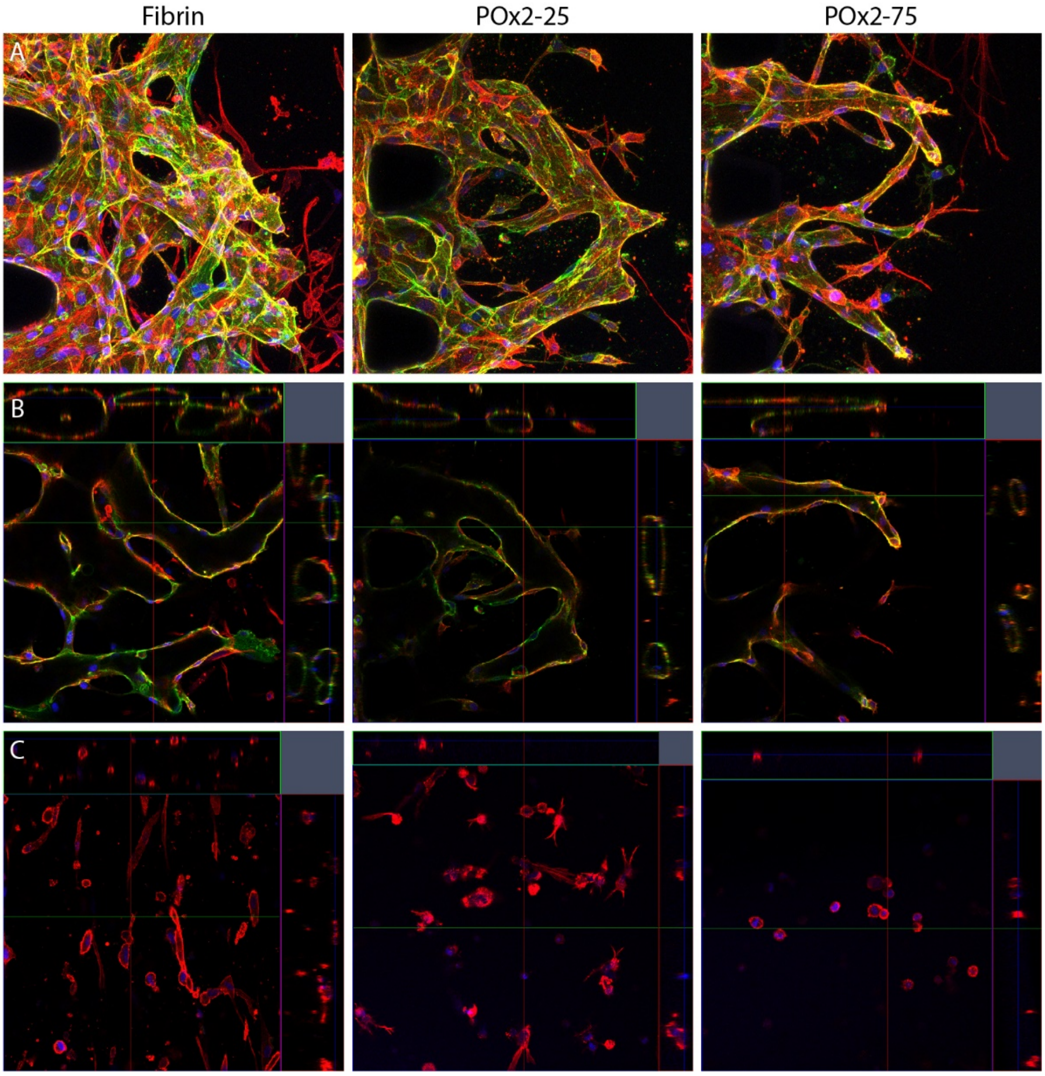

Supplementary Figure S5. Morphology of endothelial networks assembled via angiogenesis when co-cultured with MSCs (7 days of culture). A. Confocal stack of images. B. Sections, analysed using the ZEN image analysis package, displaying clear luminated structures. Scale bar 50 μm. C. Morphology of MSCs cultured in different gels after 7 days, characterised by confocal microscopy. Blue, Dapi; Red, Phalloidin; Green, CD 31. Scale bar 50 μm.

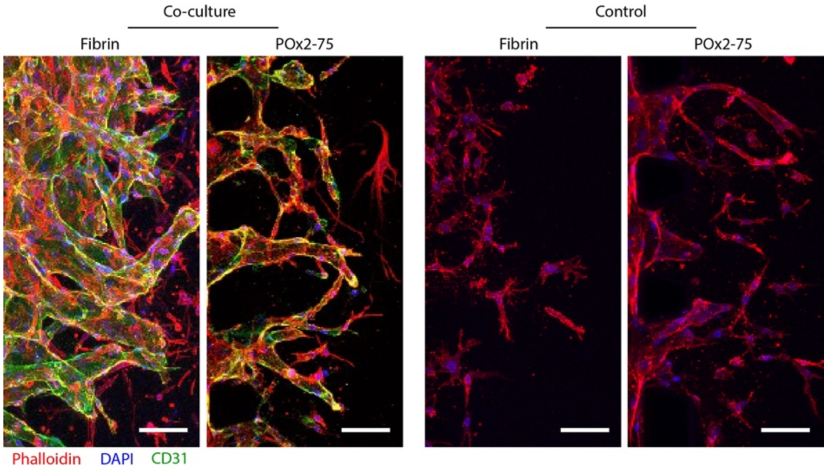

Supplementary Figure S6. Higher magnification of the morphology of endothelial networks assembled via angiogenesis in the presence of gel-loaded bmMSCs (fibrin or POx2-7 gel) or empty gels. Conditions as in Figure 4 B and C. Culture time: 7 days. Blue, Dapi; Red, Phalloidin. Scale bar 50 µm.

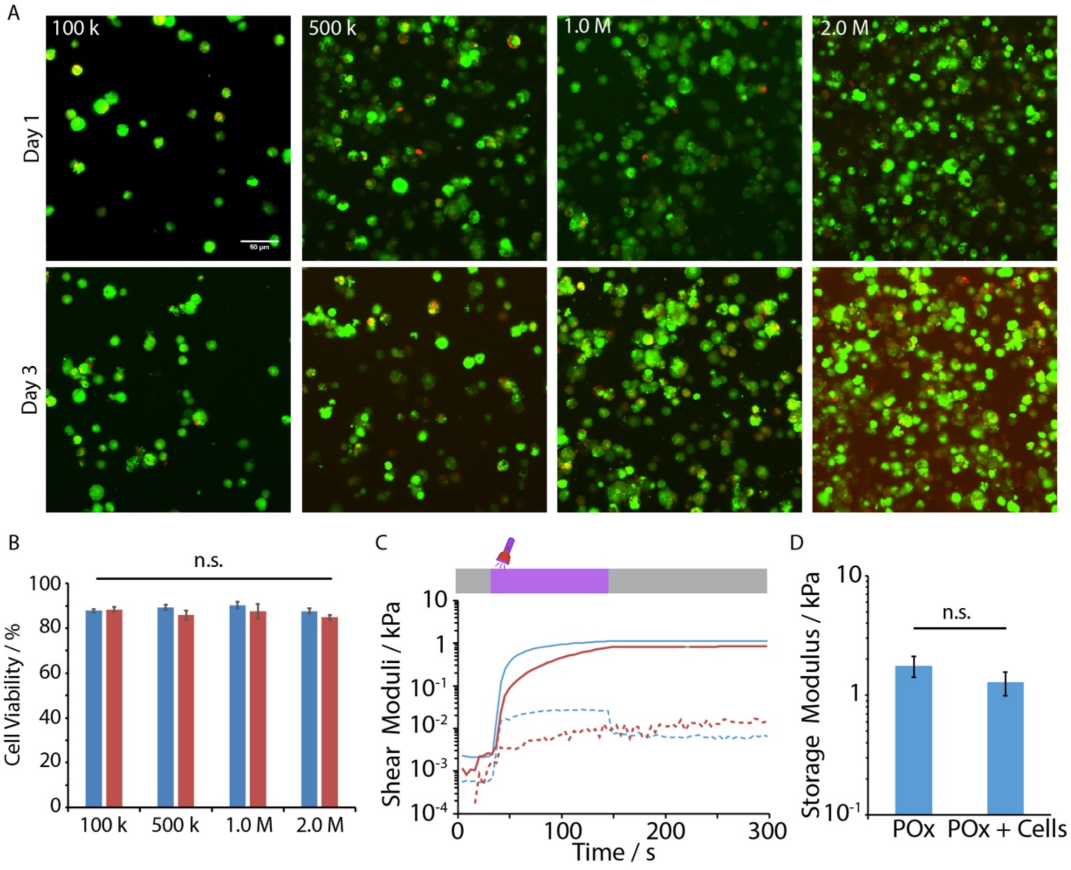

Supplementary Figure S7. A. Impact of cell density on cytocompatibility of hydrogel encapsulation on haMSCs, at days 1 and 3. Cells were encapsulated at densities of 100 k, 500 k, 1.0 million or 2.0 million cells in 100 µL of gel. Green, live; red, dead. Scale bar, 50 µm. B. Corresponding quantification. C and D. Evolution of shear moduli as a function of time during the gelation of POx2-75 hydrogels, without (blue lines) and with (red lines) cells (20.0 M cells/mL). Solid lines, storage moduli; dashed lines, loss moduli. n.s., not significant.

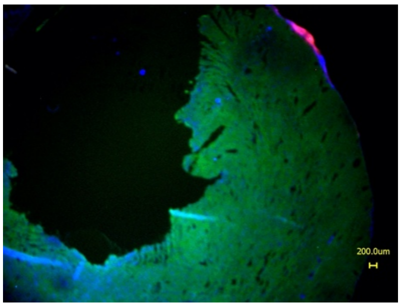

Supplementary Figure S8. Retention of ha-MSCs loaded in POx2-75 hydrogels at the surface of the epicardium, 28 days after implantation. Blue, Dapi; green, α-sarcomeric actin; red, CM-Dil labelled ha-MSCs; scale bar, 200 μm.

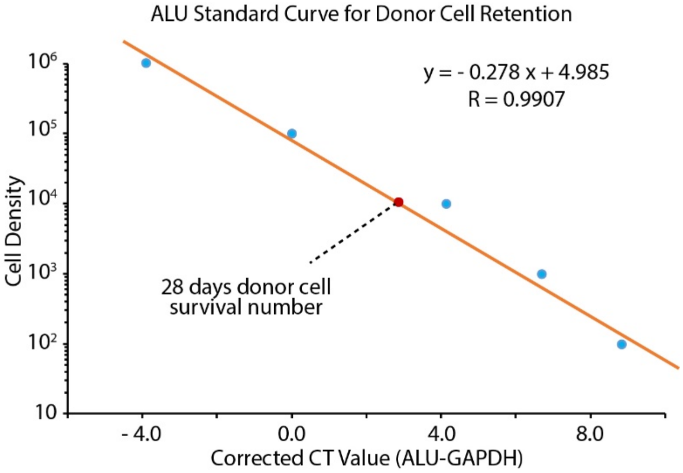

Supplementary Figure S9. ALU gene expression standard curve. (CT value normalized to GAPDH, calculated using known number of cells). 28 days after epicardial placement of POX loaded HAMSC, cell survival rate was 3.51±1.72%, calculated according to this standard curve, n=3.

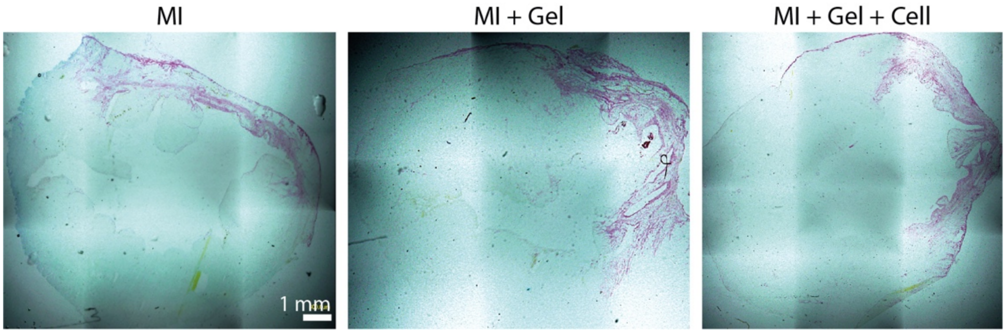

Supplementary Figure S10. Picrosirius Red staining indicating collagen deposition. Whole heart section.

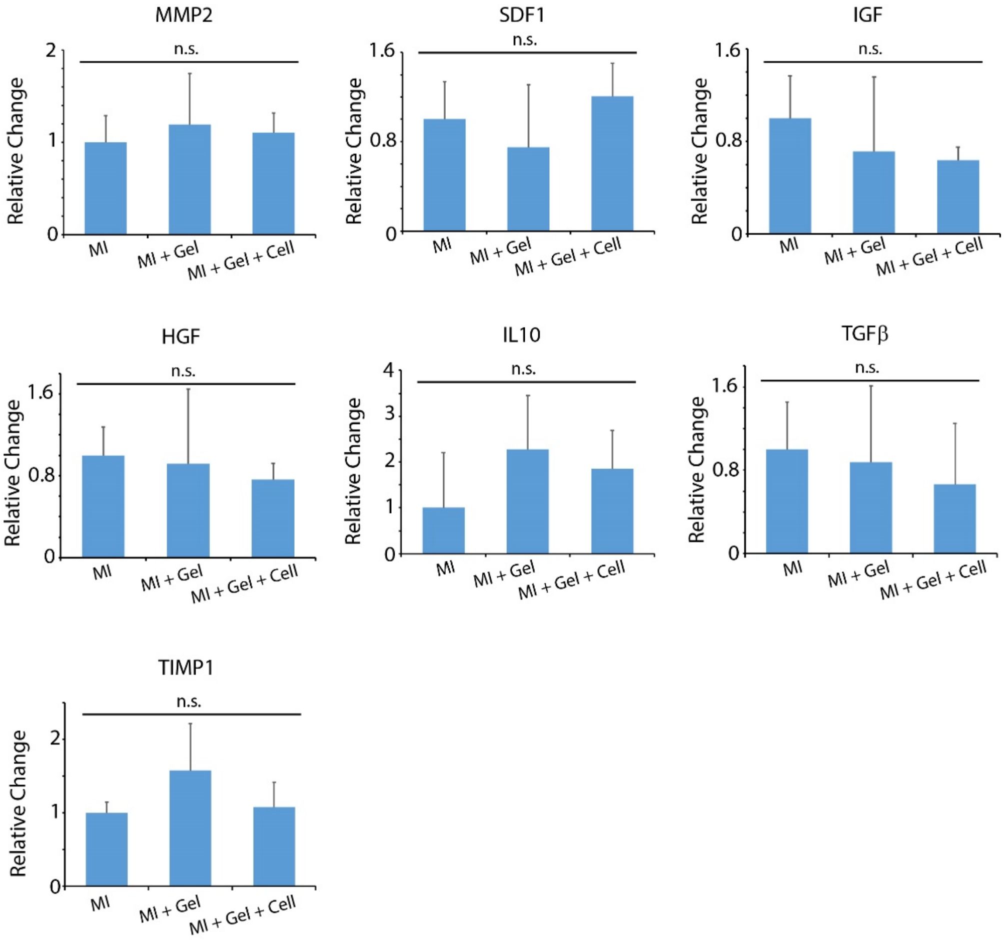

Supplementary Figure S11. Quantification of gene expression of factors associated with the secretory phenotype of MSCs promoting cardiac repair. VCMA-1 and VEGF (see Figure 8) were significantly upregulated in MI+Gel+Cell group compared to MI group, n=3 (three biological replicates for each group).

Supplementary tables

Supplementary Table S2. Statistical analysis of DNA quantification data (indicative of cell proliferation) in fibrin and polyoxazoline hydrogels, corresponding to Fig 2C. For statistical test: n.s., not significant; *P < 0.05; ***P < 0.001.

	P	Significance
Fibrin-Day4 Fibrin-Day1	0.6621	n.s.
Fibrin-Day7 Fibrin-Day1	0.02761	*
Fibrin-Day7 Fibrin-Day4	0.08268	n.s.
POX-1-50-Day4 POX-1-50-Day1	0.03343	*
POX-1-50-Day7 POX-1-50-Day1	2.61E-05	***
POX-1-50-Day7 POX-1-50-Day4	1.40E-04	***
POX-2-90-Day4 POX-2-90-Day1	0.93127	n.s.
POX-2-90-Day7 POX-2-90-Day1	0.01189	*
POX-2-90-Day7 POX-2-90-Day4	0.01767	*
POX-2-75-Day4 POX-2-75-Day1	0.51355	n.s.
POX-2-75-Day7 POX-2-75-Day1	0.02371	*
POX-2-75-Day7 POX-2-75-Day4	0.09851	n.s.
POX-2-50-Day4 POX-2-50-Day1	0.60875	n.s.
POX-2-50-Day7 POX-2-50-Day1	2.75E-05	***
POX-2-50-Day7 POX-2-50-Day4	4.31E-05	***
POX-2-25-Day4 POX-2-25-Day1	0.23618	n.s.
POX-2-25-Day7 POX-2-25-Day1	0.0279	*
POX-2-25-Day7 POX-2-25-Day4	0.27516	n.s.
POX-2-0-Day4 POX-2-0-Day1	0.76222	n.s.
POX-2-0-Day7 POX-2-0-Day1	0.01108	*
POX-2-0-Day7 POX-2-0-Day4	0.02462	*
**Comparisons at Day 7**	**P**	**Significance**
POx1-50 POx2-90	0.9343	n.s.
POx1-50 POx2-75	1	n.s.
POx1-50 POx2-50	0.95326	n.s.
POx1-50 POx2-25	1	n.s.
POx1-50 POx2-0	0.99639	n.s.
POx1-50 Fibrin	0.65033	n.s.
POx2-90 POx2-75	0.9978	n.s.
POx2-90 POx2-50	1	n.s.
POx2-90 POx2-25	0.88012	n.s.
POx2-90 POx2-0	0.66068	n.s.
POx2-90 Fibrin	0.60292	n.s.
POx2-75 POx2-50	0.95986	n.s.
POx2-75 POx2-25	1	n.s.
POx2-75 POx2-0	0.99522	n.s.
POx2-75 Fibrin	0.66863	n.s.
POx2-50 POx2-25	0.90148	n.s.
POx2-50 POx2-0	0.66438	n.s.
POx2-50 Fibrin	0.99552	n.s.
POx2-25 POx2-0	0.99951	n.s.
POx2-25 Fbrin	0.54459	n.s.
POx2-0 Fibrin	0.29896	n.s.

Supplementary Table S3. Statistical analysis of PCR results (MMP2 gene expression) presented in Fig 4A. For statistical test: n.s., not significant; ***P < 0.001.

	P	Significance
2D VS POX2-90	<0.0001	***
2D VS POX2-75	<0.0001	***
2D VS POX2-50	0.9947	n.s.
2D VS POX2-25	<0.0001	***
2D VS Fibrin	>0.9999	n.s.
POX2-90 VS POX2-75	<0.0001	***
POX2-90 VS POX2-50	<0.0001	***
POX2-90 VS POX2-25	<0.0001	***
POX2-90 VS Fibrin	<0.0001	***
POX2-75 VS POX2-50	<0.0001	***
POX2-75 VS POX2-25	<0.0001	***
POX2-75 VS Fibrin	<0.0001	***
POX2-50 VS POX2-25	<0.0001	***
POX2-50 VS Fibrin	0.9994	n.s.
POX2-25 VS Fibrin	<0.0001	***

Supplementary Table S4. Statistical analysis of PCR results (SDF1 gene expression) presented in Fig 4A. For statistical test: n.s., not significant; *P < 0.05; ***P < 0.001.

	P	Significance
2D VS POX2-90	<0.0001	***
2D VS POX2-75	<0.0001	***
2D VS POX2-50	0.7384	n.s.
2D VS POX2-25	0.0007	***
2D VS Fibrin	0.9301	n.s.
POX2-90 VS POX2-75	0.0002	***
POX2-90 VS POX2-50	<0.0001	***
POX2-90 VS POX2-25	<0.0001	***
POX2-90 VS Fibrin	<0.0001	***
POX2-75 VS POX2-50	<0.0001	***
POX2-75 VS POX2-25	<0.0001	***
POX2-75 VS Fibrin	<0.0001	***
POX2-50 VS POX2-25	0.0327	*
POX2-50 VS Fibrin	0.9983	n.s.
POX2-25 VS Fibrin	0.0124	*

Supplementary Table S5. Statistical analysis of PCR results (IL10 gene expression) presented in Fig 4A. For statistical test: **P < 0.01; ***P < 0.001.

	P	Significance
2D VS POX2-90	<0.0001	***
2D VS POX2-75	<0.0001	***
2D VS POX2-50	<0.0001	***
2D VS POX2-25	<0.0001	***
2D VS Fibrin	0.0082	**
POX2-90 VS POX2-75	<0.0001	***
POX2-90 VS POX2-50	<0.0001	***
POX2-90 VS POX2-25	<0.0001	***
POX2-90 VS Fibrin	<0.0001	***
POX2-75 VS POX2-50	<0.0001	***
POX2-75 VS POX2-25	<0.0001	***
POX2-75 VS Fibrin	<0.0001	***
POX2-50 VS POX2-25	<0.0001	***
POX2-50 VS Fibrin	<0.0001	***
POX2-25 VS Fibrin	<0.0001	***

Supplementary Table S6. Statistical analysis of PCR results (VCAM1 gene expression) presented in Fig 4A. For statistical test: n.s., not significant; ***P < 0.001.

	P	Significance
2D VS POX2-90	<0.0001	***
2D VS POX2-75	<0.0001	***
2D VS POX2-50	0.9766	n.s.
2D VS POX2-25	0.727	n.s.
2D VS Fibrin	0.8582	n.s.
POX2-90 VS POX2-75	<0.0001	***
POX2-90 VS POX2-50	<0.0001	***
POX2-90 VS POX2-25	<0.0001	***
POX2-90 VS Fibrin	<0.0001	***
POX2-75 VS POX2-50	<0.0001	***
POX2-75 VS POX2-25	<0.0001	***
POX2-75 VS Fibrin	<0.0001	***
POX2-50 VS POX2-25	0.367	n.s.
POX2-50 VS Fibrin	0.5036	n.s.
POX2-25 VS Fibrin	0.9999	n.s.

Supplementary Table S7. Statistical analysis of PCR results (IGF gene expression) presented in Supplementary Figure 4. For statistical test: n.s., not significant; ***P < 0.001.

	P	Significance
2D VS POX2-90	<0.0001	***
2D VS POX2-75	0.3712	n.s.
2D VS POX2-50	>0.9999	n.s.
2D VS POX2-25	0.3946	n.s.
2D VS Fibrin	<0.0001	***
POX2-90 VS POX2-75	<0.0001	***
POX2-90 VS POX2-50	<0.0001	***
POX2-90 VS POX2-25	<0.0001	***
POX2-90 VS Fibrin	0.9993	n.s.
POX2-75 VS POX2-50	0.4129	n.s.
POX2-75 VS POX2-25	>0.9999	n.s.
POX2-75 VS Fibrin	0.0001	***
POX2-50 VS POX2-25	0.4377	n.s.
POX2-50 VS Fibrin	<0.0001	***
POX2-25 VS Fibrin	0.0001	***

Supplementary Table S8. Statistical analysis of PCR results (VEGF gene expression) presented in Supplementary Figure 4. For statistical test: n.s., not significant; *P < 0.05; **P < 0.01.

	P	Significance
2D VS POX2-90	0.0039	**
2D VS POX2-75	0.0014	**
2D VS POX2-50	0.9747	ns
2D VS POX2-25	0.0475	*
2D VS Fibrin	0.9997	ns
POX2-90 VS POX2-75	0.9979	ns
POX2-90 VS POX2-50	0.0365	*
POX2-90 VS POX2-25	0.8743	ns
POX2-90 VS Fibrin	0.0036	**
POX2-75 VS POX2-50	0.015	*
POX2-75 VS POX2-25	0.6484	ns
POX2-75 VS Fibrin	0.0014	**
POX2-50 VS POX2-25	0.268	ns
POX2-50 VS Fibrin	0.9221	ns
POX2-25 VS Fibrin	0.0379	*

Supplementary Table S9. Statistical analysis of PCR results (TGFβ1 gene expression) presented in Supplementary Figure 4. For statistical test: n.s., not significant.

	P	Significance
2D VS POX2-90	0.9994	n.s.
2D VS POX2-75	0.9699	n.s.
2D VS POX2-50	>0.9999	n.s.
2D VS POX2-25	>0.9999	n.s.
2D VS Fibrin	0.9901	n.s.
POX2-90 VS POX2-75	0.878	n.s.
POX2-90 VS POX2-50	0.9997	n.s.
POX2-90 VS POX2-25	0.9999	n.s.
POX2-90 VS Fibrin	0.9997	n.s.
POX2-75 VS POX2-50	0.9736	n.s.
POX2-75 VS POX2-25	0.9525	n.s.
POX2-75 VS Fibrin	0.7693	n.s.
POX2-50 VS POX2-25	>0.9999	n.s.
POX2-50 VS Fibrin	0.9934	n.s.
POX2-25 VS Fibrin	0.995	n.s.

Supplementary Table S10. Statistical analysis of PCR results (HGF gene expression) presented in Supplementary Figure 4. For statistical test: n.s., not significant, *P < 0.05, **P < 0.01.

	P	Significance
2D VS POX2-90	0.4064	n.s.
2D VS POX2-75	0.005	**
2D VS POX2-50	>0.9999	n.s.
2D VS POX2-25	0.9858	n.s.
2D VS Fibrin	0.9763	n.s.
POX2-90 VS POX2-75	0.2775	n.s.
POX2-90 VS POX2-50	0.427	n.s.
POX2-90 VS POX2-25	0.7863	n.s.
POX2-90 VS Fibrin	0.876	n.s.
POX2-75 VS POX2-50	0.0074	**
POX2-75 VS POX2-25	0.0222	*
POX2-75 VS Fibrin	0.0442	*
POX2-50 VS POX2-25	0.9819	n.s.
POX2-50 VS Fibrin	0.9713	n.s.
POX2-25 VS Fibrin	>0.9999	n.s.

Supplementary Table S11. Statistical analysis of PCR results (TIMP1 gene expression) presented in Supplementary Figure 4. For statistical test: n.s., not significant; *P < 0.05.

	P	Significance
2D VS POX2-90	0.026	*
2D VS POX2-75	0.0497	*
2D VS POX2-50	0.9753	n.s.
2D VS POX2-25	0.9993	n.s.
2D VS Fibrin	0.4336	n.s.
POX2-90 VS POX2-75	0.9996	n.s.
POX2-90 VS POX2-50	0.1708	n.s.
POX2-90 VS POX2-25	0.0121	*
POX2-90 VS Fibrin	0.7645	n.s.
POX2-75 VS POX2-50	0.275	n.s.
POX2-75 VS POX2-25	0.0238	*
POX2-75 VS Fibrin	0.8956	n.s.
POX2-50 VS POX2-25	0.8935	n.s.
POX2-50 VS Fibrin	0.8777	n.s.
POX2-25 VS Fibrin	0.2725	n.s.
